# Evolutionary coupling analysis guides identification of mistrafficking-sensitive variants in cardiac K^+^ channels: Validation with hERG

**DOI:** 10.3389/fphar.2022.1010119

**Published:** 2022-10-20

**Authors:** Yihong Zhang, Amy L. Grimwood, Jules C. Hancox, Stephen C. Harmer, Christopher E. Dempsey

**Affiliations:** ^1^ School of Physiology, Pharmacology and Neuroscience, Biomedical Sciences Building, University Walk, Bristol, United Kingdom; ^2^ School of Biological Sciences, Life Sciences Building, Bristol, United Kingdom; ^3^ School of Biochemistry, Biomedical Sciences Building, University Walk, Bristol, United Kingdom

**Keywords:** misfolding, long QT syndrome, ClinVar, pathogenic, evolutionary coupling, hERG, KCNQ1

## Abstract

Loss of function (LOF) mutations of voltage sensitive K^+^ channel proteins hERG (Kv11.1) and KCNQ1 (Kv7.1) account for the majority of instances of congenital Long QT Syndrome (cLQTS) with the dominant molecular phenotype being a mistrafficking one resulting from protein misfolding. We explored the use of Evolutionary Coupling (EC) analysis, which identifies evolutionarily conserved pairwise amino acid interactions that may contribute to protein structural stability, to identify regions of the channels susceptible to misfolding mutations. Comparison with published experimental trafficking data for hERG and KCNQ1 showed that the method strongly predicts “scaffolding” regions of the channel membrane domains and has useful predictive power for trafficking phenotypes of individual variants. We identified a region in and around the cytoplasmic S2-S3 loop of the hERG Voltage Sensor Domain (VSD) as susceptible to destabilising mutation, and this was confirmed using a quantitative *LI-COR* ^®^ based trafficking assay that showed severely attenuated trafficking in eight out of 10 natural hERG VSD variants selected using EC analysis. Our analysis highlights an equivalence in the scaffolding structures of the hERG and KCNQ1 membrane domains. Pathogenic variants of ion channels with an underlying mistrafficking phenotype are likely to be located within similar scaffolding structures that are identifiable by EC analysis.

## 1 Introduction

As a result of massive genomic sequencing efforts (e.g., Taliun et al., 2021; Wang et al., 2021) a large number of natural human gene sequence variants have been identified and compiled in databases such as ClinVar (Landrum et al., 2014; Landrum et al., 2020). A large proportion of variants, especially missense sequence variants arising from single nucleotide mutations, are uncharacterized in terms of their consequences (Landrum et al., 2014; Landrum et al., 2020) and thus their classification in relation to real or potential pathogenicity is undefined. Up to 98% of missense variants across the human genome are unclassified (Frazer, Notin et al., 2021), although for well-studied disease-associated genes the proportions of unclassified variants may be in the 70–90% range (Supplementary Table S1). This underdefined classification of natural variants, especially in disease-associated genes, is a problem in circumstances where clinicians might wish to use information on potential disease causation in individuals or families that carry particular genetic variants (Ackerman, 2015). Efforts are underway either to predict (Adzhubei et al., 2010; Walsh et al., 2014; Ioannidis et al., 2016; Frazer et al., 2021) or experimentally characterize variants of genes with strong disease association [(Vanoye et al., 2018; Glazer et al., 2020; Ng et al., 2020; Oliveira-Mendes et al., 2021) for ion channel disease variants]; however, the sheer number of unclassified variants makes this a daunting task.

One way in which this bottleneck between the acquisition of genetic variant information and its useful classification for clinical value might be overcome is to predict variants expected to be either pathogenic or benign (Adzhubei et al., 2010; Walsh et al., 2014; Ioannidis et al., 2016; Frazer et al., 2021) so that experimental characterization might be focussed on a reduced set of unclassified variants. For genetic disorders in which mistrafficking of variants is a significant cause of pathogenicity, Evolutionary Coupling (EC) analysis (Fuchs et al., 2007; Kamisetty et al., 2013; Hopf et al., 2017; Nicoludis and Gaudet, 2018) should be a useful way of screening for predicted trafficking phenotypes to select variants for experimental testing. This is based on a potential causal chain that involves 1) a mutation in a variant that disrupts one or more structure stabilising interactions, 2) the reduced ability of this variant to adopt the native folded state, 3) the recognition of misfolded protein by the cellular endoplasmic reticulum (ER) quality control, and 4) the retention and degradation of the misfolded protein (mistrafficking). EC analysis identifies a set of pairwise interactions between amino acids in a protein that have resisted disruption through evolutionary time by acquisition, following single site mutations, of compensating mutations that allow recovery of interactions despite the original mutations. The method is based on the extraordinary retention of folded state structure within families of structurally homologous proteins so that pairs of evolutionary coupled residues (i.e., residue positions that have a high probability of varying in tandem) can be defined in the context of a relatively invariant folded state structure (Hopf et al., 2012).

It may seem surprising that single site mutations in a large protein (e.g., up to 1,000 amino acids in the ion channel proteins studied here) can cause a significant shift in the folded to unfolded state equilibrium and thus promote mistrafficking. However, this relationship has been established for some ion channel proteins for which mistrafficking is a significant phenotype underlying pathogenicity (Marinko et al., 2019). For two of the major cardiac voltage sensitive K^+^ ion channels that function to restore the resting membrane potential at the end of the cardiac action potential, hERG (encoded by the *KCNH2* gene) and KCNQ1, single site mutations that result in mistrafficking have been directly associated with reduced folded state stability of channel domains (Huang et al., 2018; Anderson et al., 2020). The implication is that wild type (WT) folding of these, and other membrane proteins (e.g., Schlebach et al., 2015) is thermodynamically balanced near the equilibrium between the folded (F) and unfolded (U) state. Disruption of a stabilizing interaction that results in a loss of 5–6 kJ mol^−1^ of stabilizing free energy of F relative to U has little effect on the F < - > U equilibrium for a protein with a typical folding free energy of, for example, −20 kJ mol^−1^ (e.g., proportion of folded state at equilibrium reduced from 99.95% to 99.4% for loss of 6 kJ mol^−1^ stabilizing free energy) (Huyghues-Despointes et al., 1999). However, for a protein like hERG with an apparent folding free energy, ΔG_FU_, near −4 kJ mol^−1^ [estimated from densities of mature and immature glycosylated protein bands on Western blots (Anderson et al., 2014)] loss of 6 kJ mol^−1^ of stabilizing free energy results in a reduction of the proportion of folded state from ∼ 80% to ∼ 30% with a significant effect on the proportion of correctly trafficked protein. The fact that mistrafficking of many hERG mutants can be reversed by lowering the cell culture temperature (which shifts the folding equilibrium towards the folded state) or by expressing in the presence of a hERG blocking drug that provides binding energy that favours the folded state (Anderson et al., 2014), supports the conclusion that mistrafficking of sequence variants is a consequence of misfolding resulting from loss of stabilising interactions involving the mutated amino acid residue. These considerations indicate that identifying sets of amino acid residues whose pairwise interactions contribute to folded state stability using EC analysis is likely to be useful for characterizing variants in disease states that have a significant mistrafficking phenotype.

Here we utilise published experimental trafficking data for hERG and KCNQ1 to assess the extent to which EC analysis provides a useful predictor of mistrafficking, with the expectation that the value of this analysis will be to select variants for experimental phenotyping (Hancox et al., 2020). We emphasise that our aim is not to predict pathogenicity directly but to identify so-far unclassified variants that may be predicted to mistraffic and to assess their trafficking properties. A breakdown of the numbers of hERG and KCNQ1 variants that remain to be classified is shown in Supplementary Table S1. We also compare the results of EC analysis with ClinVar classifications of variant phenotype and show that EC analysis is useful for helping to understand the structural basis of mistrafficking of ion channel proteins and as a predictor of regions of protein domains likely to be susceptible to misfolding in single site mutants. We then tested this approach by determining the trafficking phenotype of a selection of natural variants in a region of the hERG voltage sensor domain predicted by EC analysis to be susceptible to structure-disrupting mutation.

## 2 Results

### 2.1 Location of evolutionary coupled residue pairs within membrane domains

**FIGURE 1:**
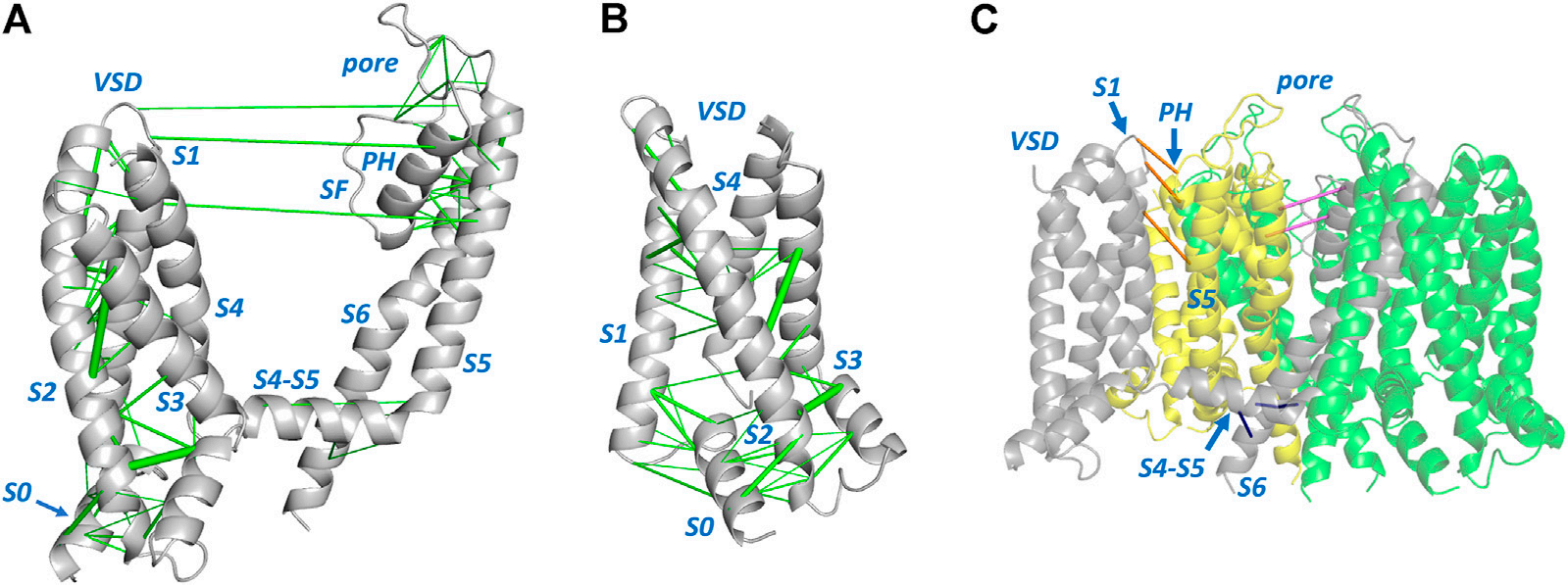
Evolutionarily coupled amino acids in the KCNQ1 channel membrane domain. **(A)** A single channel subunit from PDB:6V00 (Sun and Mackinnon, 2020) is shown with evolutionarily coupled pairs of amino acids represented by green lines connecting Cα atoms [line weight represents probability of EC pair (Hopf et al., 2019)]. **(B)** View of the KCNQ1 voltage sensor domain (VSD) illustrating EC interactions that link residues in the S0 helix with residues in helices S1 and S2. **(C)** Long distance EC interactions between residues within a single subunit **(A)** that are short range when mapped between the equivalent residues on adjacent subunits, e.g., between VSD and pore domains of adjacent subunits (orange), or between residues in adjacent pore domain subunits (pink). **(C)** also highlights EC interactions (blue) between residues in the long S4-S5 linker and S6 helix of the same subunit that are found in channel proteins with domain-swapped membrane subunits. SF: Selectivity Filter; PH: Pore Helix. In all views the side of the channel domains facing the cytoplasm is at the bottom of the figure.

**FIGURE 2:**
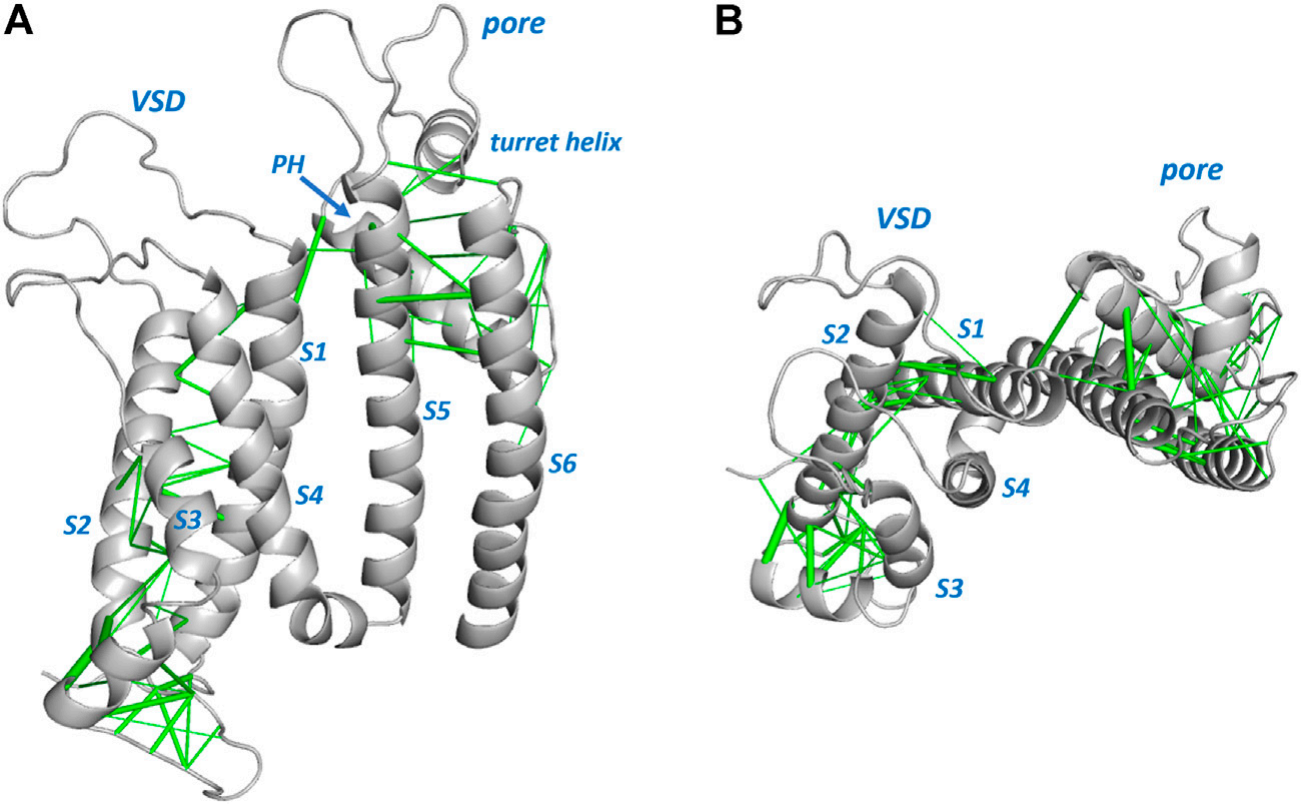
Evolutionarily coupled amino acids in the hERG channel membrane domain. A single subunit from PDB:5VA2 (Wang and Mackinnon, 2017) is shown. Sequence involving some missing atom density in extracellular loops is modeled into the structure shown. Evolutionarily coupled pairs of amino acids are represented by green lines connecting Cα atoms (line weight represents probability of EC pair), and these are shown **(A)** from the side and **(B)** looking down from the extracellular membrane surface. The latter view illustrates minimal EC interactions between the mobile S4 helix and the “scaffold” parts of the VSD involving S1-S3 (Palovcak et al., 2014). PH: Pore Helix.

Figures 1, 2 show structures of the KCNQ1 and hERG membrane domains with Evolutionary Coupled (EC) residue pairs mapped (we use “EC” to mean “Evolutionary Coupling” in the term “EC analysis” and to mean “Evolutionary Coupled” in the term “EC pair(s)”, or “EC interactions”). Evolutionary search parameters were chosen to represent sets of EC pairs corresponding to around the top 70 EC pairs in each of the hERG and KCNQ1 membrane domains. The reliability of the EC sets can be assessed in relation to the short range nature of the EC interactions in the context of the structures, and the identification of interactions identified in previous analyses of ion channel proteins. For both hERG and KCNQ1, EC interactions cluster in the bottom (cytoplasmic facing) regions of the Voltage Sensor Domains (VSDs) and the extracellular side of the pore domains. These regions constitute the “scaffold” elements of the channel proteins; i.e., the evolutionarily conserved and structurally stable regions that support the mobile regions involved in channel gating. Since EC analysis relies on the retention of conserved structure in evolutionarily related protein homologues it provides an efficient means of identifying these scaffolding regions. As previously described (Palovcak et al., 2014) the VSDs constitute scaffolds with strong interhelix coupling involving S1-S2 and S2-S3 that allows the S4 helices (limited or absent interhelical EC interactions; Figure 2B) to move in response to changes in membrane potential. EC interactions are largely absent in the lower (cytoplasmic-facing side) of the pore domains indicating that reconfiguration of the lower parts of the S5 and S6 helices associated with channel gating is incompatible with strong structural interactions.

Each of the membrane domains shows interactions involving residues at the top of the S1 helix of the VSDs and residues at the extracellular side of the pore domain (top of helix S5 and N- terminal end of the pore helices). In KCNQ1 these interactions are between the VSD and the pore domain of an *adjacent* subunit due to the domain swapped nature of KCNQ1. These interactions define anchor points between the VSDs and pore domains of voltage sensitive channels as previously described (Lee et al., 2009). If long distance EC interactions in KCNQ1 are mapped between the residues on adjacent subunits the short distance nature of these interactions is apparent (Figure 1C). The EC analysis also highlights numerous interactions involving the S0 helix previously identified as part of the scaffold structure of the KCNQ1 VSD (Huang et al., 2018) (Figure 1B), and suggests the manner in which the turret helix of hERG is physically linked to the top of the transmembrane part of the hERG pore domain (Wang and Mackinnon, 2017; Butler et al., 2020) (Figure 2). Analysis of EC interactions allows a simple distinction between domain-swapped channels (e.g., KCNQ1 and likely all members of the K_v_1-K_v_9 families of K^+^ channels) and channels that are not domain-swapped in the membrane domains (e.g., hERG and all members of the K_v_10-K_v_12 families). This is most apparent in the identification of EC interactions between the long S4-S5 linker and the S6 helix of the same subunit in KCNQ1 (Figure 1C), that are absent in non-domain-swapped channels like hERG (Figure 2). For both KCNQ1 (Figure 1) and hERG (Figure 2) high probability EC interactions were mapped onto structures of single channel states, in this case voltage-sensor-activated states existing in the absence of a membrane potential with an open pore for hERG (Wang and Mackinnon, 2017) and a closed pore for KCNQ1 (Sun and Mackinnon, 2020). The observation that high probability EC interactions map closely to the cryoEM structures indicates that the scaffold regions having high densities of EC interactions are likely to retain their structures independent of channel state, as previously described for ion channel VSDs from analysis of EC interactions (Palovcak et al., 2014).

### 2.2 Comparison of EC interactions with experimental trafficking data

Mutation of residues within the scaffold regions identified by EC analysis, that disrupt (especially) inter-helix interactions are expected to result in variants susceptible to misfolding and thus mistrafficking, and this is confirmed by assessing the location of amino acids in variants of KCNQ1 and hERG known to mistraffic (Anderson et al., 2014; Huang et al., 2018) as described below. A question is whether EC analysis can be used to assess the likely trafficking phenotype of *specific* variants, and this is explored in this section.

#### 2.2.1 KCNQ1

Huang et al. (2018) described trafficking phenotypes within a series of KCNQ1 variants involving amino acid residues in the voltage sensor domain, some of which are natural variants compiled in ClinVar (Table 1). This data set is particularly useful since the trafficking efficiency was quantitated (as a percentage of surface expression relative to WT protein) and contains several variants that have moderate to high trafficking efficiency (>40% of WT; the justification for using this value is described in the Methods). We note that some channel variants having trafficking efficiency above a threshold of 40% WT trafficking may still present a risk of pathogenicity but our aim was to assess the use of EC analysis for predicting trafficking properties of variants rather than pathogenicity.

**TABLE 1:** Comparison of EC data with experimental trafficking data from Huang et al. (2018).

Mistrafficking variants		Trafficking variants	
*match with EC*	*ClinVar*	*match with EC*	*ClinVar*
Q107H	LikPath	V100I	—
Y111C	Path	A102S	—
L114P	LikPath	T104I	—
P117L	VUS	T104S	—
Y125D	LikPath	H105L	VUS
H126L	Confl	H105N	—
E160K	VUS	H105Y	—
R174C	Path/LikPath	T118S	—
R174H	Path/LikPath	V124I	—
R174L	LikPath	F127L	VUS
W176R	Confl	A128T	—
G179S	Path	L131P	VUS
		V133I	VUS
		L134P	Confl
***mismatch***		A150T	Confl
E115G	VUS	A150V	—
G189A	—	T169M	VUS
R195P	VUS	K196T	VUS
P197S	LikPath	P197L	VUS
L236R	LikPath	V207M	LikBenign
		K218E	VUS
		I227L	LikPath
		Q234P	LikPath
		L236P	Path/LikPath
		***mismatch***	
		V106I	— ex 537
		R109L	— ex 242
		V110I	Confl ex 537
		C122Y	Confl ex 349
		V129I	VUS ex 537
		I132L	VUS ex 417
		V135A	— ex 389
		V135I	— ex 537
		A149V	— ex 319

Matches for mistrafficking variants (<40% WT trafficking) are variants involving residues found in the EC set; mismatches are residue positions absent from the EC set. For trafficking variants (>40% WT) “matches” are residue positions absent from the EC set and mismatches are residues found in EC set. ClinVar classifications are: Path: Pathogenic; Confl: Conflicting Interpretation; LikBenign: Likely Benign; LikPath: Likely Pathogenic; VUS: Variant of Uncertain Significance; - indicates variant is not in ClinVar). The number after “ex” in the trafficking mismatch set is the residue exchangeability value from Table 3 of ref (Yampolsky and Stoltzfus, 2005).

An issue with using EC analysis as a single method for assessing trafficking phenotypes is in choosing an appropriate set of EC pairs, in particular in selecting a depth of evolutionary relatedness that yields a data set likely to contain structure-stabilizing EC pairs at a high probability. The Huang et al. data set contains both mistrafficking (<40% WT) and moderate to high trafficking (>40% WT) variants and so provides a good test of the selection of structural stabilizing EC interactions while reducing “false positives” that might arise from an over-deep selection involving evolutionarily distant homologues. In other words, a good match between a high probability EC data set and experimental trafficking data requires the *presence* of amino acids found in high probability EC pairs whose variants traffic poorly as well as the *absence* of EC pairs for amino acids whose variants traffic at least moderately compared to WT. The EVcoupling analysis of both the KCNQ1 and hERG membrane domains was robust in the sense that similar sets of high probability EC interactions were found from Multiple Sequence Alignments (MSA) obtained at different depths of evolutionary selection as shown in Supplementary Table 1 for KCNQ1, and Supplementary Table 2 and Supplementary Figure S1 for hERG.

Table 1 shows a comparison of the trafficking efficiency of KCNQ1 VSD variants from Huang et al. (2018) with a set of EC pairs found reproducibly across a range of analyses obtained at different evolutionary depths. For *mistrafficking* variants a “match” occurs when the mutated residue is found with high probability in the EC set. For variants that traffic with moderate to high efficiency (>40% WT) a “match” occurs when the mutated residue is absent from the high probability EC set. In this comparison 10 of the 15 mistrafficking variant residue positions (67%) occurred at residues found in the EC set and 20 of the 28 trafficking variant residue positions (71%) were absent from the EC data set. However, several of the mismatches where moderate to efficient trafficking occurs for variants at residues predicted to make structurally-important interactions involve conservative amino acid replacements. We used an amino acid exchangeability matrix (Yampolsky and Stoltzfus, 2005) to assess the likelihood that a mutation will have a small effect on a structure-promoting interaction involving the mutated residue, taking a conservative approach that the exchangeability score should be two-thirds of the maximum possible (a score of 408 out of a maximum of 612) for highly exchangeable residue pairs. This assessment indicates that of the mismatches involving trafficking-competent variants, V106I, V110I, V129I, I132L and V135I are sufficiently conservative that important interactions involving the WT residue are likely maintained. If a conservative measure of exchangeability is incorporated into the comparison of trafficking and EC data, the EC analysis predicts 29 of 33 (88%) trafficking-competent (>40% WT) variants as unlikely to have significantly perturbed folding (i.e., likely to traffic if mistrafficking is a consequence of misfolding). EC analysis identifies amino acid *positions* likely involved in evolutionarily conserved interactions. Introducing a measure of exchangeability for specified variants allows an assessment of the agreement between EC analysis and trafficking for specific *variants* so that the numbers of variants used for comparing percentage agreements above (e.g., a match between EC analysis including residue conservation and trafficking for 29 out of 33 *variants*) is larger than the number of amino acid positions (20 out of 28 variant amino acid *positions*).

**FIGURE 3:**
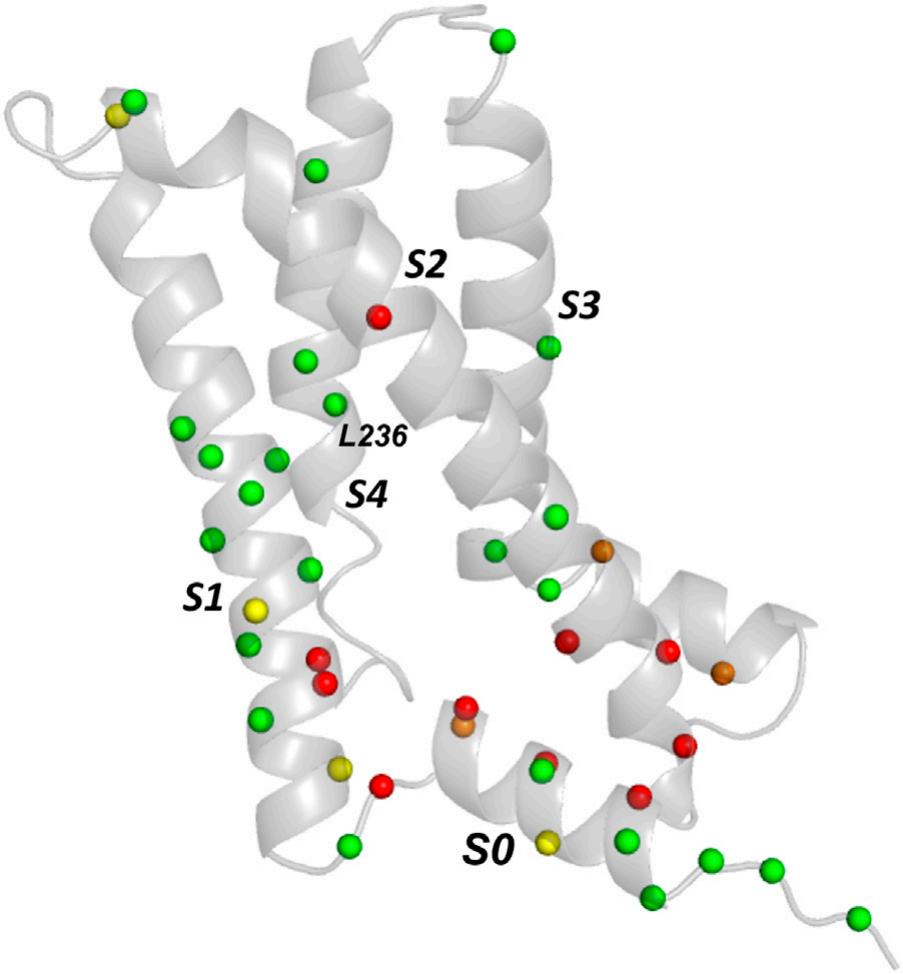
Comparison of experimental trafficking data for KCNQ1 VSD with EC analysis. Trafficking data is from (Huang et al., 2018) and the EC data for comparison is from Table 1. Green and red spheres denote matches between EC data and trafficking data. Green spheres are Cα carbons of residues having variants with moderate to good trafficking (>40% WT) that are absent from the EC data set or, if present, are conservative mutations (Table 1). Red spheres denote variants that mistraffic (<40% WT) and are present in the EC set. Orange (poor trafficking/absent from EC set) and yellow spheres (moderate to good traffickers present in the EC set with lower conservation scores) are mismatches between EC data and trafficking data.

These observations indicate that a relatively simple analysis of evolutionarily coupled residues may be useful for selecting natural channel variants for experimental phenotyping; for example, non-conservative variants in the extracellular side of the pore domain involving the pore helix and the top of the S5 and S6 helices may be predicted to misfold whereas variants involving residues in the bottom half the S5 and S6 helices are predicted to traffic competently (even if variants at these locations may have other defects, e.g., perturbation of gating). Figure 3 shows the trafficking data mapped onto a KCNQ1 VSD together with the expectation from EC analysis. Although we do not attempt an explanation for every mismatch several points can be noted (see Discussion). For example, variants in which a Pro, and possibly a Gly residue, is substituted may result in misfolding, not because these residues make important structural stabilising interactions, but because they allow particular configurations of the polypeptide chain that are lost upon replacement with a non Pro (or non Gly) residue. This might account for the mistrafficking of P197S and G189A variants despite the absence of high-probability EC interactions for these residues. Similar observations were made with hERG for Gly and Pro mutants as described below.

#### 2.2.2 hERG

We compared the hERG membrane domain trafficking data from Anderson et al. (2014) with EC data as described above for KCNQ1. Since the hERG data are not quantitative we considered several ways to compare with the EC data. The Anderson data contain many mistrafficking variants for which trafficking cannot be restored by expression at reduced temperature nor by expression with E-4031, a drug that binds at the interface of channel subunits to stabilise subunit assembly. We categorised these as “Mistrafficking Variants” in Table 2. Likewise, there is a set of variants that traffic similar to the wild type channel. Of the 28 residue *positions* in the strongly mistrafficked set 23 (82%) are found in the high probability EC set. There are only 10 residue positions in the WT trafficking set and six of these are absent from the high probability EC set as would be expected if an absence of EC interactions equated with high trafficking efficiency. If WT trafficking *variants* with highly conservative substitutions are considered as described above for KCNQ1 (V644L, M645I and M645L are conservative variants) then 9 of 12 of these variants can be considered consistent with the EC results.

**TABLE 2:** Comparison of EC data with experimental hERG trafficking data from Anderson et al. (2014).

Mistrafficking variants		Rescuable variants		Trafficking variants	
***match with EC***	***ClinVar***	***match with EC***	***ClinVar***	***match with EC***	***ClinVar***
L559H	NP	Y569H	NP	G648S	NP
A561P	Path	I571V	Confl	S649L	—
H562R	Path	E575G	NP	F656C	NP
L564P	NP	N588D	NP	G657R	NP
A565T	NP	V612L	NP	S660L	Confl
W568C	NP	T623I	NP	I662T	NP
W568R	NP	G626S	Path/LP		
G572C	NP	F627L	NP	***mismatch***	
G572D	NP	N629S	Path	Y616C ex 142	Path/LP
G572R	Path	V630A	NP	G626A ex 369	NP
G572V	Path	V630L	NP	V644L ex 537	NP
W585C	NP	N633S	Path/LP	M645I ex 612	VUS
D609G	VUS	N635D	NP	M645L ex 513	Path
D609H	NP	N635I	VUS	M645V ex 354	Path
Y611H	Path	S641F	Path		
T613M	Path/LP				
A614V	Path	***mismatch***			
S621N	Confl	L552S	Path		
S621R	NP	R582L	LP		
V625E	NP	I593R	Path		
G626D	NP	G594D	LP		
G628V	NP	P596H	Path		
N629I	NP	P596L	NP		
N629K	NP	Y597C	LP		
P632S	Path/LP	S599R	NP		
T634I	VUS	G601C	NP		
E637D	NP	L615F	LP		
E637G	NP				
E637K	Path				
K638E	NP				
K638N	NP				
F640L	Confl				
V644F	VUS				
***mismatch***					
A558E	LP				
A558P	Path				
C566S	NP				
I593G	—				
I593K	Path				
G604S	Path				
P605L	Confl				
P605S	NP				

**FIGURE 4:**
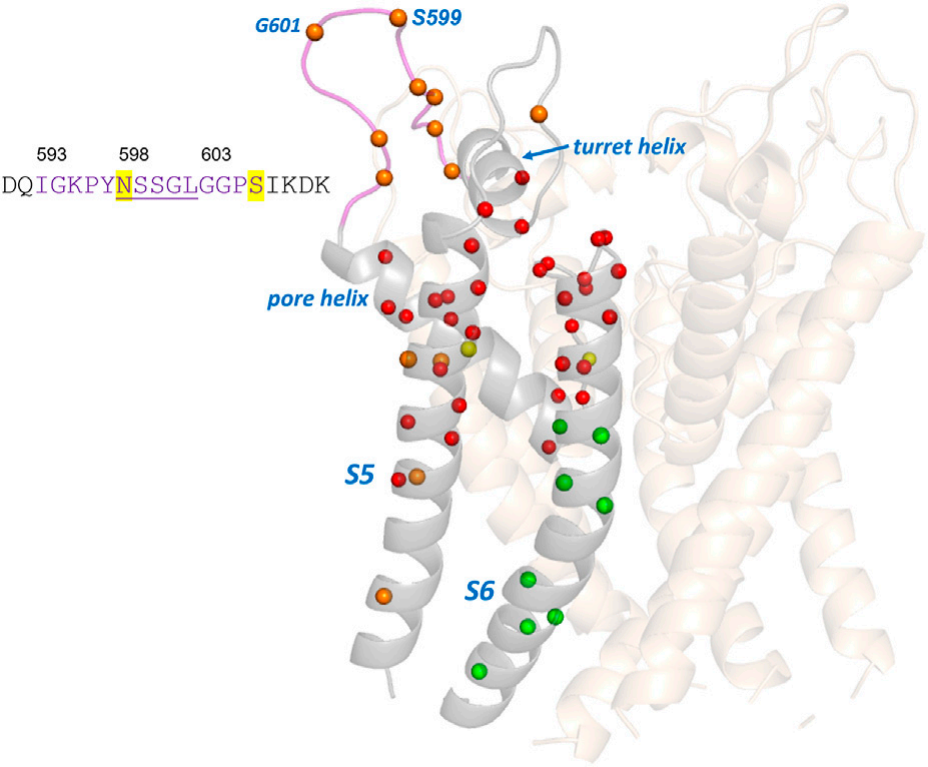
Comparison of experimental trafficking data for the hERG pore domain with EC analysis. Trafficking data is from (Anderson et al., 2014) and EC data for comparison is from Table 2. Green and red spheres are matches between EC and trafficking data. Green spheres are Cα carbons of residues having variants that traffic and are absent from the EC data set or, if present, are conservative mutations (Table 2). Red spheres denote variants that mistraffic and are present in the EC set. Orange (poor traffickers absent from EC set) and yellow spheres (moderate to good traffickers present in the EC set with lower conservation scores) are mismatches between EC data and trafficking data. The sequence of the turret loop highlights the hERG glycosylation site (N598) and a phosphorylation site (S606). Underlined amino acids are residues lacking atom density in the cryoEM structures of hERG; these residues are modeled into the structure shown.

Matches for mistrafficking variants are variants involving residues found in the EC dataset; mismatches are residue positions absent from the EC set. For trafficking variants “matches” are residue positions absent from the EC set and mismatches are residues found in EC set. Where present the ClinVar designation for the variant is given (- indicates variant is not in ClinVar; Path: pathogenic; NP: Not Provided; Confl: Conflicting Interpretation; LP: Likely Pathogenic; VUS: Variant of Uncertain Significance). The number after “ex” in the trafficking mismatches group is the residue exchangeability score (Yampolsky and Stoltzfus, 2005). Underlined variants are residues within the extracellular turret loop (see text and Figure 4).

There is a group of around 25 hERG variants in the Anderson et al. (2014) set that mistraffic but where trafficking can be restored (“rescued”) either by expression at reduced temperature or in the presence of E-4031, or both. While these phenotypes may indicate a less severe trafficking defect we have chosen not to attempt to define a reduced trafficking severity for these when comparing with the EC data since the primary expression phenotype remains a mistrafficking one. This allows us to highlight a region of mismatch between trafficking and EC analysis involving variants in an extracellular loop of the hERG pore domain turret that mistraffic, as indicated by absence of high molecular mass glycosylated bands on Western blots (Anderson et al., 2014), but involve residues that do not appear reproducibly in the EC set. This includes residues in the sequence between G594 and S606, inclusive (G594D, P596H/L, Y597C, S599R, G601C, G604S, and P605L/S; Table 2 and Figure 4). This is one of two loop regions in the hERG membrane domain (the other being the loop between the S2 and S3 helices of the voltage sensor domain; Figure 2) for which we have taken care to establish reliable sets of EC data. Thus, although occasional EC interactions involving turret loop residues are found in some EC calculations, a consistent set of EC interactions from runs involving different sequence selections containing the turret loop, was not obtained (Supplementary Table 2). We conclude that the absence of reproducible EC interactions indicates a limited number of coevolutionary relationships in the hERG turret loop that links the turret helix with the start of the pore helix (Figure 4).

A comparison of EC data with trafficking data for the hERG membrane domain is shown in Figure 4. As described above most residues in the Anderson et al. (2014) trafficking set match with expectations from EC analysis. Notably, the group of trafficking-competent variants in the lower half of the S6 helix are absent from the EC set consistent with the expectation that the regions of the pore domain that undergo significant gating-associated conformational excursions do not make structure-stabilizing interactions that would be identified by EC analysis. This does not rule out that these variants might be dysfunctional due to gating defects. Figure 4 also highlights the mismatch between EC analysis and trafficking data for the extracellular turret loop involving residues around 593 to 606. The absence of EC interactions in the turret is likely to result from the small subset of channels (KCNH family) that contains this structural element but may also indicate low structural stability. For example, the central five residues in the range N598—L602 lack atom density in the cryoEM structure consistent with structural disordering in this loop, and the high Pro, Gly, Ser content (Figure 4) is also consistent with the polypeptide having low structural propensity as described in the Discussion. However, overall, the EC analysis identifies the central core scaffold structure of the hERG pore domain involving the pore helix and extracytoplasmic halves of the S5 and S6 helices (Figure 2) consistent with the large number of mistrafficking variants in this region of the channel (Table 2; Figure 4).

### 2.3 Selecting hERG variants involving residues at the bottom of the VSD for trafficking analysis

Reproducible sets of EC interactions involving the S2-S3 loop at the bottom of the VSD (Figure 2) were obtained from EC runs covering a range of different sequences around the loop and from analyses at a range of evolutionary depths (Supplementary Table 2; Supplementary Figure S1). We conclude that the EC interactions involving the S2-S3 loop represent true coevolutionary relationships between residues in this part of the VSD, which therefore constitutes a region strongly predicted to be sensitive to structure-destabilising mutations. Based on EC analysis we selected 11 variants, 10 of which are natural hERG variants, involving residues in and around the S2-S3 loop for trafficking analysis. This set represents each residue position that is both involved in reproducible high-probability EC coupling involving loop residues and which also had a variant in ClinVar. These are listed in Table 3 along with the location within the VSD structure, their ClinVar classifications and EVE (Frazer et al., 2021), PolyPhen-2 (Adzhubei et al., 2010) and PROVEAN (Choi and Chan, 2015) predictions. K407A was included as a residue making several EC interactions (Figure 8) although this has not been identified as a natural variant. We also included A614V (Tanaka et al., 1997) and L615F (Napolitano et al., 2005) as controls for variants with well-established mistrafficking phenotypes (Anderson et al., 2006; Anderson et al., 2014).

**TABLE 3:** hERG VSD variants selected for trafficking phenotyping based on EC analysis.

*Variant*	*Location*	*ClinVar*	*EVE*	*PolyPhen-2*	*PROVEAN*
I400N	pre-S1	NP	Path	Probably Damaging	Deleterious
H402R	pre-S1	VUS	Path	Possibly Damaging	Deleterious
K407A	S1	—	Path	Probably Damaging	Deleterious
R472P	S2-S3 loop	LP	Path	Probably Damaging	Deleterious
T473P	S2-S3 loop	LP	Path	Probably Damaging	Deleterious
T474I	S2-S3 loop	Path	Path	Probably Damaging	Deleterious
Y475C	S2-S3 loop	NP	Path	Probably Damaging	Deleterious
V476I	S2-S3 loop	VUS	Uncertain	Benign	Neutral
V483F	S2-S3 loop	VUS	Path	Probably Damaging	Deleterious
R488C	S3	VUS	Uncertain	Probably Damaging	Deleterious
H492L	S3	VUS	Path	Probably Damaging	Deleterious
A614V	pore helix	Path	Path	Probably Damaging	Deleterious
L615F	pore helix	LP	Path	Probably Damaging	Deleterious

ClinVar classifications (accessed 12 March 2022) are LP: Likely pathogenic; NP: Not Provided; Path: Pathogenic; VUS: Variant of Uncertain Significance; -: not in Clinvar. EVE predictions were accessed on 12 March 2022. Polyphen-2 and PROVEAN evaluations were made on 1 April 2022.

### 2.4 Trafficking phenotypes of selected variants in the hERG VSD

**FIGURE 5:**
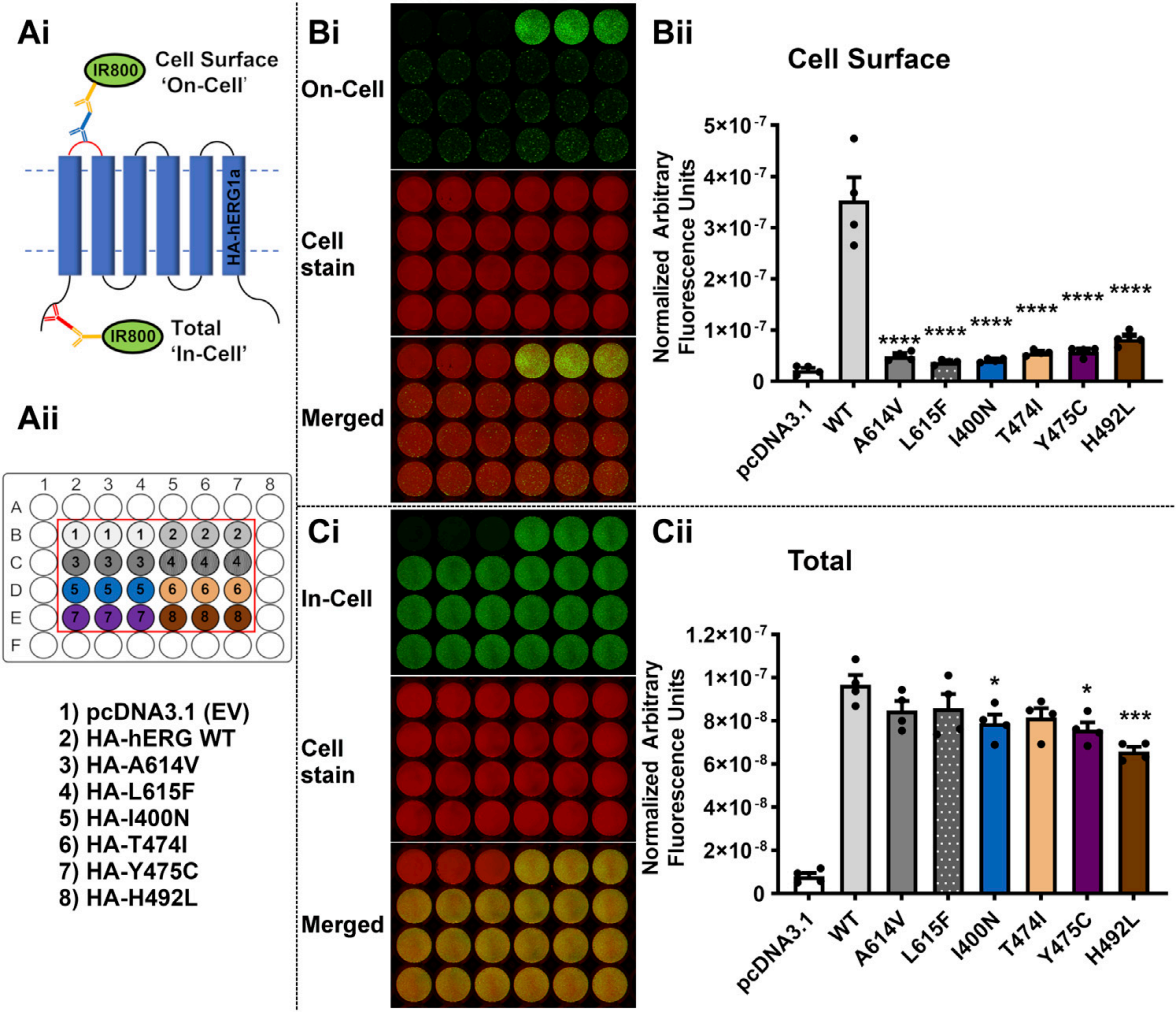
The variants I400N, T474I, Y475C and H492L severely perturb trafficking of the hERG channel. **(Ai)**: Diagram depicting the experimental setup for Cell Surface (‘On-Cell’) and Total channel protein detected (Total) (‘In-Cell’) Western assays. **(Aii)**: Diagram of the assay design and key for transfection conditions for the assays displayed in **(Bi,Ci)**. EV = empty vector; WT = Wild-Type. **(Bi):** ‘On-Cell’ Cell Surface hERG channel expression of the test variants I400N, T474I, Y475C and H492L compared to the WT channel and positive control variants A614V and L615F. Representative ‘On-Cell’ Cell Surface hERG channel expression assay images are shown. The upper panel (green channel) shows the ‘On-Cell’ Cell Surface signal for hERG expression. The middle panel (red channel) represents cells stained with WGA-680 and the bottom panel is the merged image. **(Bii)** Quantified Cell Surface expression levels of the WT channel, positive controls, and test variants. **(Ci)** ‘In-Cell’ Total cellular hERG channel protein for the test variants I400N, T474I, Y475C and H492L compared to the WT channel and positive control variants A614V and L615F. Representative ‘In-Cell’ Total hERG channel protein assay images are shown. The upper panel (green channel) shows the ‘In-Cell’ Total hERG channel protein detected. The middle panel (red channel) represents cells stained with WGA-680 and the bottom panel is the merged image. **(Cii)** Quantified Total channel protein values for the WT channel, positive controls, and test variants. Data are presented as (mean ± SEM) from four independent repeats [individual data points are displayed in **(Bii,Cii)**]. Statistical analyses were performed using one way analysis of variance (ANOVA) and Bonferroni’s multiple comparison. Asterisks indicate a significant difference from the corresponding WT value (**** = *p* < 0.0001; *** = *p* < 0.001; ** = *p* < 0.005; * = *p* < 0.05).

**FIGURE 6:**
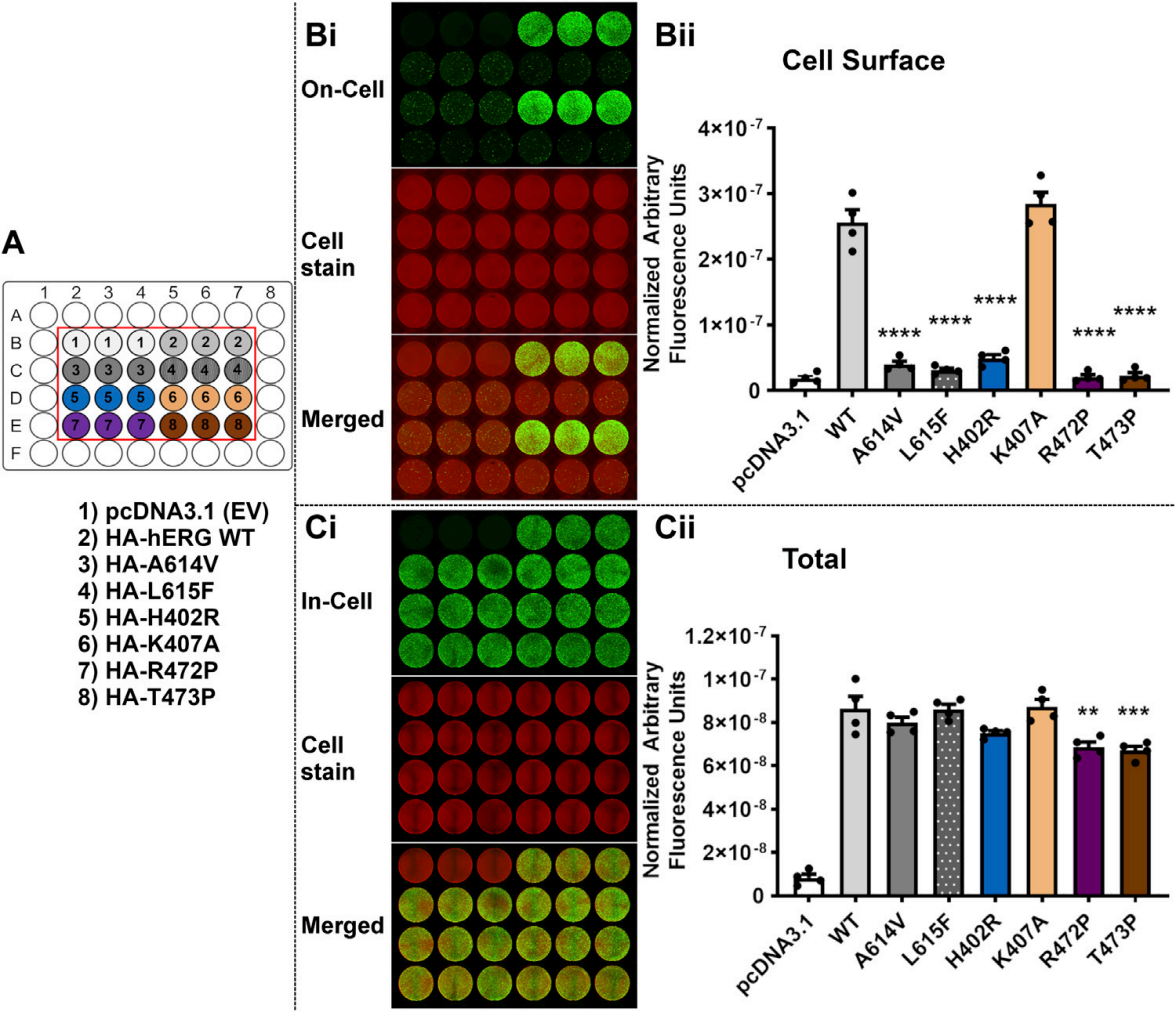
Variants H402R, R472P, T473P but not K407A severely perturb trafficking of the hERG channel. Trafficking assay for four hERG VSD variants H402R, K407A, R472P, T473P with WT hERG and controls (A614V and L615F). A detailed description of the panels can be found in the legend to Figure 5. Data are presented as (mean ± SEM) from four independent repeats [individual data points are displayed in **(Bii,Cii)**]. Statistical analyses were performed using one way analysis of variance (ANOVA) and Bonferroni’s multiple comparison. Asterisks indicate a significant difference from the corresponding WT value (**** = *p* < 0.0001; *** = *p* < 0.001; ** = *p* < 0.005).

**FIGURE 7:**
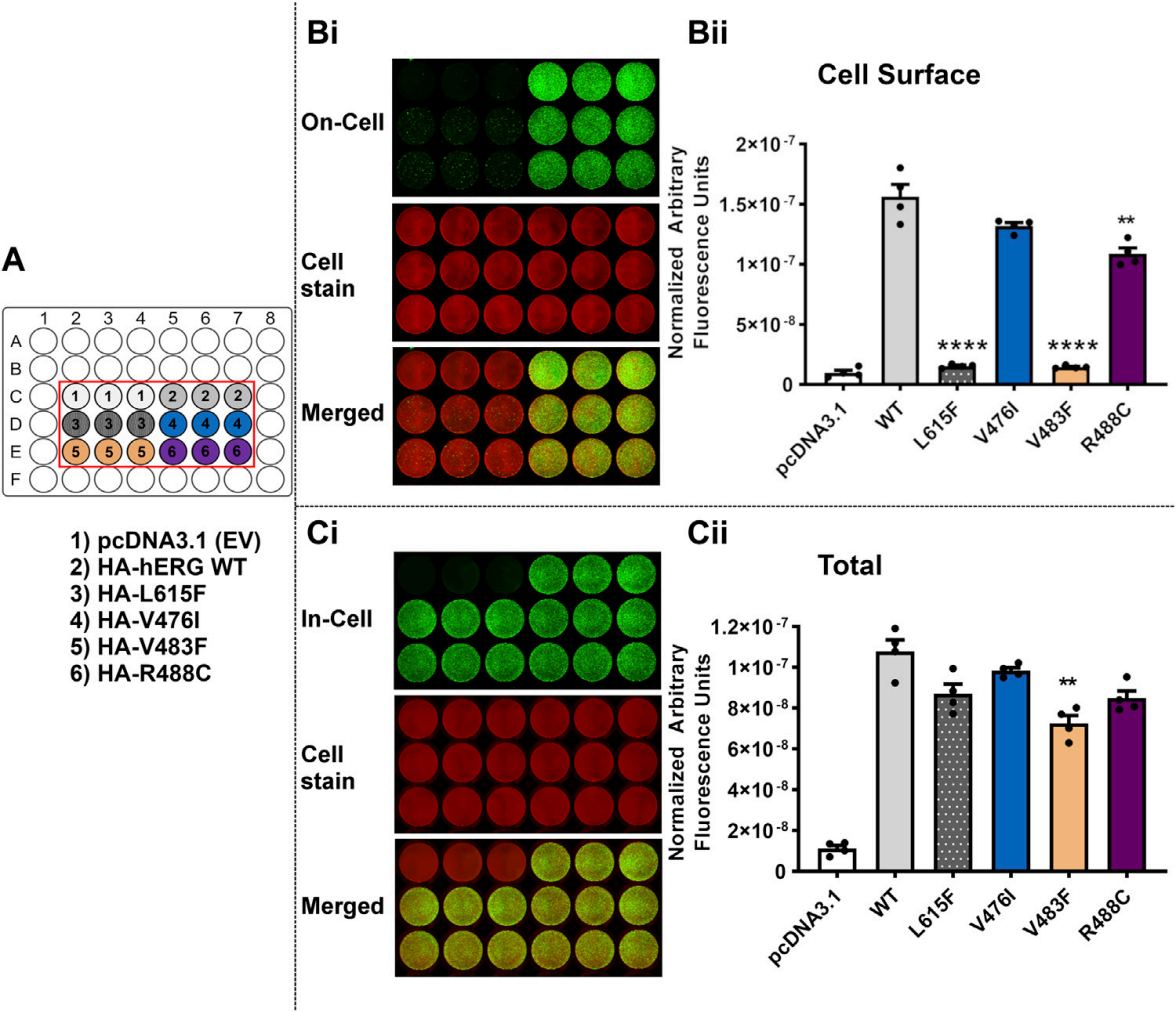
Variant V483F, but not V476I and R488C, severely perturbs trafficking of the hERG channel. Trafficking assay for three hERG VSD variants V476I, V483F and R488C with WT hERG and control (L615F). A detailed description of the panels can be found in the legend to Figure 5. Data are presented as (mean ± SEM) from four independent repeats [individual data points are displayed in **(Bii,Cii)**]. Statistical analyses were performed using one way analysis of variance (ANOVA) and Bonferroni’s multiple comparison. Asterisks indicate a significant difference from the corresponding WT value (**** = *p* < 0.0001; ** = *p* < 0.005).

The *LI-COR®* based trafficking assay described previously (Al-Moubarak et al., 2020) allows a direct and quantitative measure of the expression of hERG at the membrane surface in transiently transfected HEK-293 cells. For each of the selected variants listed in Table 3, both ‘In-Cell’ (total channel protein detected in the cell) and ‘On-Cell’ (protein expressed at cell membrane surface) values were measured and compared with WT and empty vector. The 11 variants tested were grouped into three sets for experimental convenience. One or both of the mistrafficking variants A614V and L615F was included as positive controls in each experimental set. Each assay was repeated on four separate occasions with data points for individual repeats shown in the bar charts in Figures 5–7.

Figure 5A illustrates a schematic of the assays, with representative ‘On-Cell’ and ‘In-Cell’ expression shown in Panels B and C, respectively; the arrangements of WT, controls and tested variants in the wells are indicated in the ‘key’ in Aii. Visual inspection of the fluorescence levels in panel B shows marked reductions in cell surface expression compared to WT for two positive controls A614V and L615F and the four tested variants from Table 3 (I400N, T474I, Y475C and H492L). The normalized data for cell surface expression (mean ± SEM) are shown in the bar chart in Figure 5Bii. Compared with WT, both positive controls and the four variants showed greatly attenuated cell surface hERG expression (*p* < 0.0001 for all 6 variants versus WT). When conducting multiple comparison to pcDNA3.1 (empty vector; EV control), all variants showed no statistical difference (*p* > 0.05), except for WT, which is *p* < 0.0001 versus pcDNA3.1. The ‘In-Cell’ assay (Figure 5C) shows high levels of expression for all variants with only I400N, Y475C and H492L showing statistically significant reductions compared to WT (*p* < 0.05 for I400N and Y475C; *p* < 0.001 for H492L). These results indicate that reduced variant expression contributes, at most, only a small amount to the striking reductions in cell surface expression seen for these variants.

Figure 6 shows equivalent trafficking results for a second set of variants. Along with the mistrafficking controls A614V and L615F, three of the tested variants H402R, R472P and T473P showed marked attenuation of cell surface expression (*p* < 0.0001 versus WT), but for K407A there was no significant difference from WT surface expression (*p* > 0.05 versus WT). When conducting multiple comparison against the empty vector (EV) control (pcDNA3.1), the values for the three test variants (H402R, R472P and T473P) were not significantly different (*p* > 0.05 compared with pcDNA3.1). In the In-Cell assay, only R472P and T473P showed significant, but modest, reductions in total detected channel protein compared to WT levels [∼77% and 79% of WT respectively (Table 4)].

**TABLE 4:** Summary of trafficking results for hERG voltage sensor domain variants and controls.

	**Cell surface channel expression (% of WT, mean ± SE)**	**Total channel detected (% of WT, mean ± SE)**
**A614V** [pathogenic] (8)	8.8 ± 0.5	89.9 ± 2.9
**L615F** [pathogenic] (12)	4.8 ± 0.7	89.9 ± 5.0
**I400N** [VUS] (4)	6.1 ± 1.1	80.0 ± 1.2
**H402R** [VUS] (4)	13.0 ± 1.5	87.3 ± 6.5
**K407A** [not in ClinVar] (4)	114.7 ± 12.1	103.0 ± 8.5
**R472P** [likely pathogenic] (4)	0.9 ± 0.5	79.1 ± 6.9
**T473P** [VUS] (4)	1.9 ± 0.7	76.7 ± 6.1
**T474I** [pathogenic] (4)	10.4 ± 1.2	82.9 ± 2.6
**Y475C** [VUS] (4)	11.0 ± 1.4	77.0 ± 3.4
**V476I** [VUS] (4)	84.9 ± 6.8	91.0 ± 4.3
**V483F** [VUS] (4)	3.1 ± 0.8	64.9 ± 6.5
**R488C** [VUS] (4)	68.3 ± 4.3	78.0 ± 8.9
**H492L** [VUS] (4)	18.9 ± 2.7	65.7 ± 4.8

VUS: Variant of Uncertain Significance. The value in parenthesis in column one is the number of independent replicates.

Figure 7 shows the results of trafficking assays for the third set of variants. Like the mistrafficking control L615F, V483F showed greatly attenuated cell surface trafficking (*p* < 0.0001 compared with WT) to levels that were not significantly different from empty vector (*p* > 0.05 versus pcDNA3.1). The hERG variant R488C had significantly reduced cell surface expression (*p* < 0.005 versus WT). Cell surface expression of V476I was not significantly different from WT levels (*p* > 0.05). All of the variants showed high levels of total channel protein in the ‘In-Cell’ assay with only V483F showing a significant reduction compared with WT expression (*p* < 0.005) [∼65% of WT level (Table 4)].

The expression and trafficking data for all variants tested are compiled in Table 4 as percentages relative to WT. In calculating these percentages, the pcDNA3.1 empty vector values (background signal) for both total protein detected in the ‘In-Cell’ assay and cell surface expression was subtracted. As previously described using Western blot analysis of immature and mature glycosylated hERG protein, both positive controls A614V (Anderson et al., 2006) and V615F (Anderson et al., 2014) showed severely attenuated cell surface expression in our assay (less than 10% of WT cell surface expression; Table 4). For each of these controls the In-Cell measurements show that they are synthesized at levels similar to the WT protein, so that the greatly attenuated ‘On-Cell’ expression is a result of the inability of the synthesized protein to traffic to the cell membrane. Similar conclusions can be made for variants I400N, H402R, R472P, T473P, T474I and Y475C which showed cell surface expression less than 15% of WT levels while retaining high total expression levels (at least 75% of WT). The large attenuation of cell surface expression of V483F and H492L was associated with moderate reductions in total protein detected. Variants K407A and V476I showed high levels of total protein and cell surface expression that were not significantly different from WT levels. The cell surface expression of R488C was significantly but moderately reduced compared to WT levels (∼68% of WT) without significant differences in total channel protein detected.

Of the set of natural ‘Long QT’ variants studied here, some trafficking data have been obtained in previous studies and this allows us to compare data obtained with a quantitative assay that directly assesses total protein and cell surface expression with more qualitative analyses obtained using Western blots to detect relative levels of immature and mature glycosylated protein. Phan et al. showed that I400N and H402R variants had reduced membrane surface expression compared to WT protein when expressed in HEK-293 cells at 37°C (Phan et al., 2017). Our data confirm this finding and demonstrate that attenuation of cell surface expression of these variants is severe. Phan et al. (2017) were also able to measure gating kinetics for the K407A mutant expressed in *Xenopus* oocytes at 17°C indicating significant levels of expression and trafficking in this system, and our data confirm that this mutation has little effect on hERG cell surface expression in mammalian cells at 37°C. Likewise, for T473P (Liu et al., 2013) and T474I (Anderson et al., 2006), each first identified in LQTS patients [(Liu et al., 2013) and (Tanaka et al., 1997) for T473P and T474I, respectively], Western blot analysis of glycosylation status showed attenuated production of mature protein. Our data confirm that cell surface expression of these hERG channel variants is severely attenuated and associated with levels of total protein production that are only slightly reduced compared to WT levels, again demonstrating severe mistrafficking phenotypes. We are not aware of published data describing trafficking in mammalian cells of the other natural variants in our set although the absence of mature (fully glycosylated) hERG Y475C on overexpression in HEK cells has been reported in an abstract (Lin et al., 2011).

The moderate reductions in total protein detected in the ‘In-Cell’ assay for several variants (I400N, R472P, T473P, Y475C, V483F and H492L; Figures 5–7; Table 4) are considerably smaller than those reported in a recent study of KCNQ1 trafficking, where levels of total protein were reduced to below 30% of WT levels for a several variants (Huang et al., 2018). In that study, co-expression with a proteasome inhibitor, MG132, resulted in recovery of total protein expression but had only a limited effect on enhancing surface expression of poorly trafficking variants, indicating that KCNQ1 variants with folding deficiencies that result in attenuated trafficking to the cell surface are more efficiently targeted for proteasomal degradation (Huang et al., 2018). These observations may represent a difference in the processing of hERG and KCNQ1 mistrafficking variants. For example, Western blots of glycosylation status indicate that the immature form of hERG (the 135 molecular mass, partially glycosylated band that is a marker for the non-trafficked form) is detected at a qualitatively similar level to the wild-type channel for a range of LQT2 variants [e.g., Figure 1A of Perry et al. (2016)]. In other words, mistrafficking hERG variants may have longer lifetimes before protein recycling, compared to mistrafficking KCNQ1 variants. However direct comparison of hERG and KCNQ1 mistrafficking under equivalent assay conditions is likely to be required to assess this possibility. Like KCNQ1, proteolytic degradation of poorly folding hERG variants may be responsible for the moderate attenuation of total protein detected in several of the variants reported here although it cannot be ruled out that some variants are synthesised marginally less efficiently than WT hERG. If cell surface protein is expressed as a proportion of total protein detected (rather than as a proportion of WT cell surface protein), to give an estimate of “apparent trafficking efficiency” this indicates that R488C may traffic normally (Supplementary Figure S2; Supplementary Table S4), although the trafficking assay indicates that presentation of this variant at the cell surface is significantly reduced whether the primary defect is due to mistrafficking, reduced protein production or enhanced degradation or a combination of these.

**FIGURE 8:**
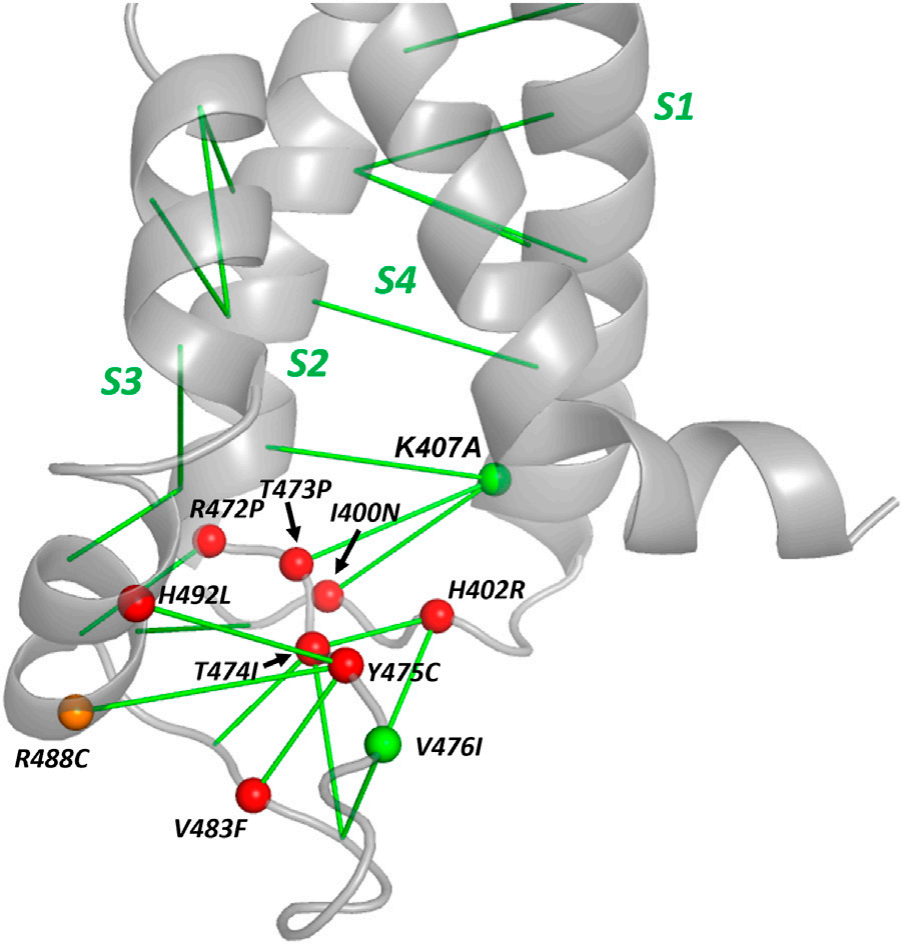
A folding-sensitive region around the cytoplasmic S2-S3 loop of the hERG voltage sensor domain. The backbone structure on the cytoplasmic side of the VSD is shown with the location of tested variants identified with a sphere on the Cα atom. Red and orange spheres are variants with severe or moderate reductions in cell surface expression, respectively and green spheres indicate variants with WT trafficking (Table 4). EC interactions are represented with green lines connecting Cα atoms. The structural contexts of residues K407 and R488 are illustrated in Supplementary Figure S3.

A summary of the location of the hERG variants within the voltage sensor domain, the EC interactions that were used as a basis of selecting these variants and their trafficking phenotypes is shown in Figure 8. This highlights the region in and around the S2-S3 loop at the bottom of the hERG VSD as a folding-sensitive region with non-conservative mutations likely to cause mistrafficking in homotetrameric channels. Surprisingly, K407A traffics normally despite K407 1) being evolutionarily coupled with three residues [I400, N470 (Zhou et al., 1999) and T473; Table 4; Figure 8; Supplementary Figure S3] that are themselves sensitive to mistrafficking in non-conservative variants, and 2) making structurally defined interactions (Armstrong et al., 2016) with these residues (especially N470 and T473) in the hERG structure (Supplementary Figure S3). It was previously shown that the K407A mutation results in a large attenuation of the deactivation kinetics of hERG when expressed in oocytes (Phan et al., 2017). This observation together with the apparent absence of stabilizing interactions involving the K407 is consistent with an interpretation that the K407 side chain may interact with the membrane potential to undergo voltage-dependent reconfiguration associated with gating. For the moderately cell surface expressed variant R488C, the R488 side chain is oriented away from its evolutionary coupled partners (Y475 and N477; Supplementary Figure S3) in the cryoEM structure (Wang and Mackinnon, 2017). However, interactions of the R488 guanidine group with the Y475 and N477 side chains with geometries previously identified for Arg side chain interactions (Armstrong et al., 2016) can be achieved by reorientation of the side chain.

### 2.5 Discussion

The potential relationship between the identification of evolutionarily coupled residues as indicators of structure-stabilising interactions and the loss of stabilising interactions in missense mutations in mistrafficking-sensitive proteins, is broadly supported by the results presented here. As previously shown for membrane channel proteins EC analysis identifies the “scaffold” elements of structure (Palovcak et al., 2014), and it is these parts of structure that are expected to be sensitive to structure-destabilising missense mutations and thus mistrafficking. EC analysis identifies a region at the bottom of the hERG voltage sensor domain in and around the S2-S3 linker as a potential scaffold element of the channel (Figure 2), supporting movement of the mobile S4 helix in response to changes in the membrane potential. We have assessed the trafficking efficiency of 11 single nucleotide mutations in this region, 10 of which are natural variants and all of which involve residues predicted to be sensitive to structure-destabilising mutation. Of these, eight natural variants have severe mistrafficking phenotypes and one variant (V476I) which traffics with close to WT efficiency is a conservative mutation whose trafficking efficiency is not unexpected. These results confirm the prediction that the region at the bottom of the hERG VSD around the S2-S3 loop is susceptible to mistrafficking in missense variants and likely to contribute to scaffolding of the voltage sensor domain. The S2-S3 loop which is common to the broader family of cyclic nucleotide binding homology domain channels (James and Zagotta, 2018), may also make interactions with cytoplasmic domains of the channel.

Two missense mutants, one a natural variant (R488C) and one (K407A) at a residue lacking natural variants in ClinVar to date, conform less well to the expectation that a non-conservative mutation at a residue making significant EC interactions within a folding-sensitive structural element should have a mistrafficking phenotype. These mismatches are of themselves interesting since they highlight additional considerations that can be made when assessing the potential of evolutionary and physicochemical approaches to variant trafficking (and pathogenicity) prediction as described below.

#### 2.5.1 Assessing missense variants in ion channel proteins using evolutionary couplings

The ability of EC analysis to make discrete predictions about the trafficking phenotype of *specific* variants is moderate: around 80% predictability when the exchangeability of amino acid substitutions is considered, as indicated by comparison of published trafficking data with EC predictions (Tables 1, 2) and including our trafficking data of variants selected from EC predictions (Table 4; Figure 8). The nature of our comparisons of EC data with experimental trafficking data in which all data (experimental and EC data) is converted to binary descriptors (traffic/mistraffic for experimental data; absent/present in high-probability EC datasets) is a potential limitation of our approach. However, we have not attempted to optimize the predictability beyond incorporating amino acid exchangeability for conservative mutations, and we consider mismatches between EC analysis and trafficking phenotypes to be instructive of structural features of the ion channels. Consideration of mismatches highlights the expectation that substitution of Pro (especially) and Gly residues may result in structural perturbation leading to misfolding and mistrafficking even if these residues lack apparent coupling to other residues. For both KCNQ1 (Table 1) and hERG (Table 2) two out of the five amino acid positions in which mistrafficking variants are absent from the EC sets involve Pro or Gly. For helical membrane proteins these amino acids are important in supporting flexibility in helix-based segments required for folding (Shelar and Bansal, 2016). The analysis also highlights the context-dependence of trafficking-disrupting amino acid variation. For example, variants involving L236 in the KCNQ1 VSD S4 helix either mistraffic (L236R) or traffic with moderate efficiency (L236P) (Huang et al., 2018) (Table 1). Since L236 lies within the mobile S4 helix this residue is not expected to make significant structure stabilising interactions within the VSD as suggested by the absence of EC’s involving this residue in our data sets. In this case trafficking variability may indicate the ability of the translocon to insert a helical element containing a neutral amino acid substitution (L236P) more efficiently into the membrane than a positively charged substitution (L236R) (Hessa et al., 2007). A more detailed analysis of the local environment of a residue within the folded structure is likely to be an important consideration for the predictability of structural perturbation in mutants for *globular* domains of proteins and we have found, for example, that the matches between EC data sets and trafficking phenotypes for variants in the cytoplasmic N- and C-terminal domains of hERG are less good than for the membrane domains (unpublished results), probably because individual buried residues in a globular domain are less likely to make discrete pairwise interactions with other residues. This contrasts with the membrane domains of eukaryotic membrane proteins where the importance of interactions between adjacent transmembrane helices (Hopf et al., 2012; Nicoludis and Gaudet, 2018) is a factor in the predictability of EC analysis for assessing the effects of missense mutations as described here.

It should be noted that EC analysis might also give rise to some spurious identification of potential interacting residues at amino acid positions that have varied in parallel within a deep evolutionary period represented within the Multiple Sequence Alignment but which have become unassociated in the extant version of the protein. In these cases, apparently coupled residues may no longer make substantial contributions to folding state stability leading to a mismatch between prediction and experimental trafficking phenotype. This might account for the retention of moderate trafficking in R488C since the R488 side chain is not in contact with its evolutionarily coupled partners (Supplementary Figure S3), at least in the available cryoEM structure (PDB: 5VA2) (Wang and Mackinnon, 2017). The WT trafficking of K407A is more surprising since the K407 side chain interacts with favourable geometries with at least two of its evolutionarily coupled partners in the hERG cryoEM structure (Supplementary Figure S3), and the three amino acids to which it is coupled (I400, N470 and T473) are themselves sensitive to destabilising (and mistrafficking) mutations [Table 4; (Zhou et al., 1999) for N470D]. The implication is that these interactions involving K407 do not make a net favourable contribution to the stability of the hERG VSD.

The most prominent mismatch between EC datasets and experimental trafficking data involves residues in the loop in the hERG turret that links the turret helix with the N-terminal end of the pore helix (Table 2; Figure 4). Seven residues within this loop have variants with significant mistrafficking phenotypes as assessed by glycosylation status (Anderson et al., 2014) and three variants P596H, G601S and G604S are classified as “pathogenic” in ClinVar. We were unable to identify a consistent set of EC residue pairs involving residues in this loop (Supplementary Table 2), and this may relate to the fact that the KCNH sub-family of channel proteins which contain an extracellular turret in the pore domain is relatively small and evolutionarily distinct (Li et al., 2015; James and Zagotta, 2018). Accounting for this by analyzing EC sets obtained from shallower evolutionary searches to focus on more closely related hERG homologues is limited by the loss of acceptable probabilities for EC’s obtained from MSAs with fewer sequences. However, it is also possible that the inability to obtain reliable EC interactions for this loop indicates an absence of structure-stabilizing interactions involving these residues. It seems unlikely that residues within the region lacking atom density in cryoEM structures of hERG (N598-L602, inclusive; Figure 4) make structure-stabilizing interactions, and 10 of the 13 residues in the loop (residues 594–606) are amino acids highly represented in datasets of disordered sequence (Deiana and Giansanti, 2010), especially Ser, Gly and Pro. This loop contains the evolutionarily conserved glycosylation site (N598) that is a marker for hERG trafficking and that is found in all members of the KCNH family of K_v_ channels, and a phosphorylation site (S606) that may also contribute to hERG trafficking (Jiang et al., 2018). Mistrafficking variants in this loop might therefore relate to loss of sequence requirements for specific recognition by cellular trafficking machinery rather than the loss of interactions that stabilise channel structure. Although these possibilities are not easily distinguished at present, further focus on this part of the channel should be useful for a fuller understanding of hERG trafficking and variant pathogenicity; any interpretations should accommodate the observation that G601S mistrafficking is rescuable by hERG pore blockers like E-4031 (Ficker et al., 2002). The recently described EVE analysis for predicting variant pathogenicity (Frazer et al., 2021) also fails to predict pathogenic variants in this loop (P596H and G601S have pathogenic classifications in ClinVar but are predicted benign by EVE, and pathogenic ClinVar variants involving I593 at the start of the loop have uncertain EVE predictions); PolyPhen-2 and PROVEAN also fail to predict P596H and G601S as pathogenic. Although more direct measures of folding highlight the relationship between mistrafficking and folded state stability for mistrafficking hERG PAS domain variants (Anderson et al., 2020) and KCNQ1 VSD variants (Huang et al., 2018), analysis of the hERG turret loop highlights the possibility that mistrafficking of variants may also occur for reasons other than destabilization of folded state structure.

Although we are using EC analysis for assessing likely trafficking phenotypes rather than predicting pathogenicity, the similarities with pathogenicity predictions (Tables 3, 4), including the difficulties associated with predicting variant phenotypes in the hERG turret loop (Figure 4), are not unexpected. Prediction methods based on sequence, evolutionary and structural analysis overlap conceptually with EC analysis and these methods are expected to be most successful in identifying variants likely to cause loss of function (LOF) of marginally stable proteins through misfolding and mistrafficking. For example, application of computational prediction methods (including PolyPhen2, and PROVEAN) to LQTS genes has been shown to be moderately successful for predicting pathogenic KCNQ1 (LQTS type 1) and hERG (KCNH2; LQTS type 2) variants but less so for cardiac Na^+^ channel variants (SCN5A; LQTS type 3) (Leong et al., 2015) where LQTS (type 3) pathogenicity is predominantly associated with a gain of function (GOF) phenotype, with gating deficiencies (perturbed inactivation) in otherwise folding-competent pathogenic SCN5A variants (Giudicessi et al., 2018). New channel-specific computational tools [e.g., (Heyne et al., 2020; Zhang et al., 2020; Phul et al., 2022)] may improve variant phenotype predictability, although experimental phenotyping is still required to improve confidence for clinical decision making. While EC analysis may be informative in structural analysis of Na^+^ channels it is not expected to be useful in itself for predicting SCN5A LQTS type 3 variant phenotypes. We anticipate that the most useful application of EC analysis is in identifying likely misfolding-sensitive regions of proteins and the selection of variants for experimental phenotyping as described here for hERG. EC analysis should also be useful for identifying proteins or protein domains in which misfolding may be the dominant phenotype underlying pathogenicity but where this has not been established. In these instances, known pathogenic variants are expected to cluster within the “scaffolding” regions identified using EC analysis. Pathogenic variants that fall outside of these scaffolding regions are more likely to have an underlying gating defect.

#### 2.5.2 Conclusion

Our results confirm the use of Evolutionary Coupling analysis for identifying scaffolding elements of ion channel proteins likely to harbour mistrafficking variants but also demonstrate its value in selecting natural channel variants for experimental phenotyping. The analysis highlights a trafficking-sensitive region in and around the S2-S3 loop of the hERG VSD which we have validated experimentally, and illustrates that trafficking sensitive regions of the hERG and KCNQ1 membrane domains are broadly similar including a likely role for residues preceding the S1 helix (identified as the S0 helix in KCNQ1 (Huang et al., 2018) in structure stabilization. It is likely that all KCNQ1 and hERG paralogues that have mistrafficking as a prominent mechanism underlying pathogenic phenotypes will have a similar distribution of mistrafficking variants in equivalent scaffolding regions to those described here. The quantitative trafficking data obtained here and elsewhere (Kanner et al., 2018; Kozek et al., 2020; Ng et al., 2022) will be useful for assessing the reliability of more detailed predictive methods for variant phenotypes and to establish clearer relationships between trafficking phenotypes and pathogenicity in channel variants. In this regard a more comprehensive analysis of the hERG VSD S2-S3 variants involving trafficking phenotypes and channel gating properties when coexpressed with wild type channels to reflect the heterozygous state encountered clinically, and the potential for rescue of trafficking in mistrafficking variants, will be important.

## 3 Methods and materials

### 3.1 EVcouplings data analysis

EC data for regions in the hERG and KCNQ1 membrane domains were obtained from the EVcouplings server (Hopf et al., 2019) although similar analyses were made using data from Gremlin (Kamisetty et al., 2013). To identify reproducible sets of high probability EC data, multiple sequences were submitted encompassing the specific sequence region of interest and runs with a range of different Bitscores were performed to assess the effects of analyzing Multiple Sequence Alignments (MSA) with shallower or deeper evolutionary relationships with the submitted sequence. To account for some drop-off in identification of EC interactions involving residues near the edges of a sequence of interest, submitted sequences were extended by up to ten amino acid residues at the N- and C-termini. Apart from assessing the effects of varying Bitscores, default EVcouplings parameters were used (PseudoLikelihood Maximation and UniRef90 database). Since our aim was to identify potential structure stabilising interactions largely involving interhelix or intraloop residue pairs, only long range EC interactions (*i*, *i* + *n*, where *n* > 5) were retained in order to remove a large number of *i*, *i* + 3 and *i*, *i* + 4 couplings that are typical of helical structure. For comparing experimental trafficking data with EC data separate EVcouplings runs were submitted using either the full membrane domain sequence or the sequence of the domain that contains the experimental trafficking data (the VSD sequence for KCNQ1 and the pore domain sequence for hERG; see Figures 3, 4). The sequence ranges for these EVcouplings runs are listed in Supplementary Table S3. EVcouplings runs were repeated initially with Bitscores of 0.4, 0.5 and 0.6 (equivalent to medium to shallow evolutionary searches in EVcouplings designation) to give a range of submissions with different sets of homologues within the MSA’s. EVcouplings output was selected that had ranges of Seq/Len (number of sequences in MSA/length of sequence) between 10 and 60 and only EC pairs with a probability of 0.9 or greater as specified in the EVcouplings output was retained for analysis. Supplementary Table 1 (KCNQ1) and Supplementary Table 2 (hERG) illustrate the reproducibility of EC sets obtainable within a range of submitted sequences and Bitscores. For assessing the reproducibility of EC interactions in and around the hERG VSD S2-S3 loop, EVcouplings outputs from an extended set of sequence ranges was obtained and this analysis is illustrated in Supplementary Figure S1. Structural figures were prepared using PyMOL Molecular Graphics System, Version 2.0 Schrödinger, LLC.

### 3.2 Trafficking data

Trafficking data for hERG and KCNQ1 variants were obtained from the Supplementary Material accompanying refs (Anderson et al., 2014) and (Huang et al., 2018), respectively. Data for KCNQ1 is tabulated as percentage of wild type trafficking in (Huang et al., 2018); we chose a value of 40% wild type trafficking as a cut-off to make a binary distinction between trafficking deficient and trafficking competent variants. This value was chosen based on the thermodynamic argument outlined in the Introduction that mistrafficking results from a significant shift in the folding equilibrium towards the unfolded state so that a meaningful thermodynamic destabilization should correspond to at least a few kilojoules per mole in terms of a shift in the folding-unfolding equilibrium. The majority of variants in the Huang et al. data set have percentage trafficking efficiencies either much lower or much greater than 40% of wild-type and so the particular value of the cut-off is not critical to our analysis.

Trafficking data for hERG tabulated in (Anderson et al., 2014) are non-quantitative and based on the densities of immature and mature gel bands in Western blots. We classed the set of variants that mistraffic and are not rescued by expression at lowered temperature or in the presence of E-4031 as strongly mistrafficked, and the set that traffic similar to wild type as trafficking variants (Table 2). The set of mistrafficking but rescuable variants are classed as rescuable in Table 2. For comparison with EC data in Figure 4, the trafficking data are divided into mistrafficking (strongly mistrafficked and rescuable) and trafficking.

### 3.3 Mutagenesis

All mutations were generated using the QuikChange Site-Directed Mutagenesis Kit (200519, Agilent Technologies, Stockport, United Kingdom) as in our previous reports (Zhang et al., 2011; Zhang et al., 2016; Al-Moubarak et al., 2020). Supplementary Table S2 lists all complementary oligonucleotide primers for each mutant containing the mutation sites which were synthesized by Merck (Merck Life Science United Kingdom Limited, Dorset, United Kingdom). These were used in a PCR reaction (95°C for 1 min, 60°C for 1 min, 68°C for 16 min for 18 cycles) together with HA-tagged WT hERG 1a plasmid expression vector as the DNA template. The HA-tagged hERG1a construct (Apaja et al., 2013) was a gift from Professor Alvin Shrier (McGill University, Canada) and contains the reference hERG1a (*KCNH2*) sequence (NM_000238.3, NCBI) with an extracellular HA tag inserted between the S1 and S2 domains. A DpnI digest of the PCR mix was then performed for 3 h at 37°C. Competent DH5α *Escherichia coli* (18263012, Life Technologies, Paisley, United Kingdom) were transformed using standard procedures. The mutations were confirmed by sequencing of the entire open reading frame (Eurofins MWG Operon, Ebersberg, Germany).

### 3.4 Cell culture and transfection

HEK-293 cells (European Collection of Cell Cultures, Porton Down, United Kingdom) were maintained at 37°C, 5% CO_2_ in Dulbecco’s minimum essential medium with Glutamax-1 (DMEM; Gibco, Paisley, United Kingdom). This was supplemented with 10% fetal bovine serum (Zhang et al., 2011; Zhang et al., 2016). For On/In-Cell Western assays HEK-293 cells were cultured on poly-l-lysine coated (P4707, Merck Life Science United Kingdom Limited, Dorset) 48 well Nunc plates (150687, ThermoFisher Scientific, Paisley, United Kingdom) using only the centre 24 wells (the outside wells contained Phosphate Buffered Saline (PBS)). Near confluent cells were transfected using Lipofectamine 2000 (11668027, ThermoFisher Scientific, Paisley, United Kingdom) on the day following cell plating according to the manufacturer’s instructions, with a total of 1 µg of vector DNA per well. Each transfection condition was performed in triplicate (to provide technical replicates).

### 3.5 ‘On-Cell’ (cell surface expression) Western assay

This assay was used to quantify HA-hERG channel expression at the plasma membrane (PM) and full experimental details can be found in (Al-Moubarak et al., 2020). In brief, 48 hours after transfection, cells were incubated with primary antibody (mouse monoclonal anti-HA antibody (H9658, Merck Life Science United Kingdom Limited, Dorset)) diluted 1:1,000 at 4°C for 1 h. After washing (at 4°C), cells were fixed in 3.7% formaldehyde (252549, Merck Life Science United Kingdom Limited, Dorset) for 20 min at room temperature (RT). After fixation and washing, cells were incubated with Wheat Germ Agglutinin (WGA) 680 Alexa Fluor (W32465, Life technologies, Paisley, United Kingdom) at 5 μg/ml in Hanks’ Balanced Salt Solution (HBSS) for 10 min. After washing, a secondary antibody (anti-mouse IgG (H + L) (DyLight 800 conjugate) (5257S, Cell Signaling Technology, Leiden); 1:1,000 dilution) was added for 1 h. After three final washes, assay plates were imaged using a *LI-COR* ^®^ Odyssey CLx imaging system.

### 3.6 ‘In-Cell’ (total hERG protein) Western assay

This assay was used to quantify the total amount of HA-hERG channel detected in fixed and permeabilized cells and full experimental details can be found in (Al-Moubarak et al., 2020). In brief, 48 hours after transfection, cells were fixed in 3.7% formaldehyde for 20 min at RT, followed by staining with WGA-680 using 5 μg/ml diluted in HBSS for 10 min. After fixation, cells were permeabilized using 0.1% Triton X-100 (Triton X100, Merck Life Science United Kingdom Limited, Dorset). After permeabilization, cells were blocked for 30 min before adding the primary anti-hERG antibody (sc-377388, Santa Cruz Biotechnology, Dallas U.S.A; 1:1,000 dilution) for 1 h. After washing, the cells were incubated with secondary antibody (anti-mouse IgG (H + L) (DyLight 800 conjugate) (5257S, Cell Signaling Technology, Leiden); 1:1,000 dilution) for 1 h. After three final washes, assay plates were imaged using a *LI-COR* ^®^ Odyssey CLx imaging system.

### 3.7 Analysis and normalization of On/In-Cell Cell Western assay data

A normalisation by the sum approach (Degasperi et al., 2014) was adopted. Raw arbitrary fluorescence intensities (700 and 800 channel values) obtained from the *LI-COR®* Odyssey CLx machine were exported into Excel (Microsoft). On a well-by-well basis the raw intensities for the 800 channel were normalised to that of 700 channel (WGA-680 cell stain) and the mean values for the triplicate technical replicates were calculated. These were normalised to the total summed signal value of the 800 channel for that assay to obtain the normalized arbitrary fluorescence for each experimental condition. The mean ± standard error of the mean (SEM) were calculated from the four independent assay repeats as presented in the bar graphs in Figures 5–7.

To calculate the % of WT for cell surface channel expression and % of WT total channel protein detected values described in the main text and Table 4, we first subtracted the normalized arbitrary fluorescence units of the pcDNA3.1 transfected condition (empty vector transfection - non-specific staining) from all conditions and then normalized to the WT control value for each set. Mean ± SEM was then calculated from the 4 independent assay repeats (as listed in Table 4).

### 3.8 Data analysis and statistics

Data are presented as mean ± SEM. Statistical analysis was performed on the normalized arbitrary fluorescence from all conditions in that set using ordinary One-Way ANOVA with Bonferroni post-hoc test for multiple comparison as appropriate. Details of the statistical test results are given alongside *p* values either in the main text or relevant Figure legend.

## Data Availability

The original contributions presented in the study are included in the article/Supplementary Material, further inquiries can be directed to the corresponding authors.

## References

[B1] Ackerman M. J. (2015). Genetic purgatory and the cardiac Channelopathies: Exposing the variants of uncertain/unknown significance issue. Heart Rhythm 12, 2325–2331. 10.1016/j.hrthm.2015.07.002 26144349

[B2] Adzhubei I. A. Schmidt S. Peshkin L. Ramensky V. E. Gerasimova A. Bork P. (2010). A method and server for predicting damaging missense mutations. Nat. Methods 7, 248–249. 10.1038/nmeth0410-248 20354512 PMC2855889

[B3] Al-Moubarak E. Zhang Y. Dempsey C. E. Zhang H. Harmer S. C. Hancox J. C. (2020). Serine mutation of a conserved threonine in the hERG K^+^ channel S6-pore region leads to loss-of-function through trafficking impairment. Biochem. Biophys. Res. Commun. 526, 1085–1091. 10.1016/j.bbrc.2020.04.003 32321643 PMC7237882

[B4] Anderson C. L. Delisle B. P. Anson B. D. Kilby J. A. Will M. L. Tester D. J. (2006). Most LQT2 mutations reduce Kv11.1 (hERG) current by a class 2 (Trafficking-Deficient) mechanism. Circulation 113, 365–373. 10.1161/CIRCULATIONAHA.105.570200 16432067

[B5] Anderson C. L. Kuzmicki C. E. Childs R. R. Hintz C. J. Delisle B. P. January C. T. (2014). Large-scale mutational analysis of Kv11.1 reveals molecular insights into type 2 long QT syndrome. Nat. Commun. 5, 5535. 10.1038/ncomms6535 25417810 PMC4243539

[B6] Anderson C. L. Routes T. C. Eckhardt L. L. Delisle B. P. January C. T. Kamp T. J. (2020). A rapid solubility assay of protein domain misfolding for pathogenicity assessment of rare DNA sequence variants. Genet. Med. 22, 1642–1652. 10.1038/s41436-020-0842-1 32475984 PMC7529867

[B7] Apaja P. M. Foo B. Okiyoneda T. Valinsky W. C. Barriere H. Atanasiu R. (2013). Ubiquitination-dependent quality control of hERG K^+^ channel with acquired and inherited conformational defect at the plasma membrane. Mol. Biol. Cell. 24, 3787–3804. 10.1091/mbc.E13-07-0417 24152733 PMC3861077

[B8] Armstrong C. T. Mason P. E. Anderson J. L. Dempsey C. E. (2016). Arginine side chain interactions and the role of arginine as a gating charge carrier in voltage sensitive ion channels. Sci. Rep. 6, 21759. 10.1038/srep21759 26899474 PMC4761985

[B9] Butler A. Helliwell M. V. Zhang Y. H. Hancox J. C. Dempsey C. E. (2020). An update on the structure of hERG. Front. Pharmacol. 10, 1572. 10.3389/fphar.2019.01572 32038248 PMC6992539

[B10] Choi Y. Chan A. P. (2015). Provean web server: A tool to predict the functional effect of amino acid substitutions and indels. Bioinformatics 31, 2745–2747. 10.1093/bioinformatics/btv195 25851949 PMC4528627

[B11] Degasperi A. Birtwistle M. R. Volinsky N. Rauch J. Kolch W. Kholodenko B. N. (2014). Evaluating strategies to normalise biological replicates of western blot data. Plos One 9, E87293. 10.1371/journal.pone.0087293 24475266 PMC3903630

[B12] Deiana A. Giansanti A. (2010). Predictors of natively unfolded proteins: Unanimous consensus score to detect a twilight zone between order and disorder in generic datasets. Bmc Bioinform. 11, 198. 10.1186/1471-2105-11-198 PMC2877690 20409339

[B13] Ficker E. Obejero-Paz C. A. Zhao S. Brown A. M. (2002). The binding site for channel blockers that rescue misprocessed human long QT syndrome type 2 ether-A-GoGo-related gene (hERG) mutations. J. Biol. Chem. 277, 4989–4998. 10.1074/jbc.M107345200 11741928

[B14] Frazer J. Notin P. Dias M. Gomez A. Min J. K. Brock K. (2021). Disease variant prediction with deep generative models of evolutionary data. Nature 599, 91–95. 10.1038/s41586-021-04043-8 34707284

[B15] Fuchs A. Martin-Galiano A. J. Kalman M. Fleishman S. Ben-Tal N. Frishman D. (2007). Co-evolving residues in membrane proteins. Bioinformatics 23, 3312–3319. 10.1093/bioinformatics/btm515 18065429

[B16] Giudicessi J. R. Wilde A. A. M. Ackerman M. J. (2018). The genetic architecture of long QT syndrome: A critical reappraisal. Trends cardiovasc. Med. 28, 453–464. 10.1016/j.tcm.2018.03.003 29661707 PMC6590899

[B17] Glazer A. M. Wada Y. Li B. Muhammad A. Kalash O. R. O'Neill M. J. (2020). High-throughput reclassification of SCN5A variants. Am. J. Hum. Genet. 107, 111–123. 10.1016/j.ajhg.2020.05.015 32533946 PMC7332654

[B18] Hancox J. C. Stuart A. G. Harmer S. C. (2020). Functional evaluation of gene mutations in long QT syndrome: Strength of evidence from *in vitro* assays for deciphering variants of uncertain significance. J. Congenit. Heart. Dis. 4, 6. 10.1186/s40949-020-00037-9

[B19] Hessa T. Meindl-Beinker N. M. Bernsel A. Kim H. Sato Y. Lerch-Bader M. (2007). Molecular code for transmembrane-helix recognition by the Sec61 translocon. Nature 450, 1026–1030. 10.1038/nature06387 18075582

[B20] Heyne H. O. Baez-Nieto D. Iqbal S. Palmer D. S. Brunklaus A. May P. (2020). Predicting functional effects of missense variants in voltage-gated sodium and calcium channels. Sci. Transl. Med. 12, eaay6848. 10.1126/scitranslmed.aay6848 32801145

[B21] Hopf T. A. Colwell L. J. Sheridan R. Rost B. Sander C. Marks D. S. (2012). Three-dimensional structures of membrane proteins from genomic sequencing. Cell. 149, 1607–1621. 10.1016/j.cell.2012.04.012 22579045 PMC3641781

[B22] Hopf T. A. Green A. G. Schubert B. Mersmann S. Scharfe C. P. I. Ingraham J. B. (2019). The EVcouplings Python framework for coevolutionary sequence analysis. Bioinformatics 35, 1582–1584. 10.1093/bioinformatics/bty862 30304492 PMC6499242

[B23] Hopf T. A. Ingraham J. B. Poelwijk F. J. Scharfe C. P. Springer M. Sander C. (2017). Mutation effects predicted from sequence Co-variation. Nat. Biotechnol. 35, 128–135. 10.1038/nbt.3769 28092658 PMC5383098

[B24] Huang H. Kuenze G. Smith J. A. Taylor K. C. Duran A. M. Hadziselimovic A. (2018). Mechanisms of KCNQ1 channel dysfunction in long QT syndrome involving voltage sensor domain mutations. Sci. Adv. 4, eaar2631. 10.1126/sciadv.aar2631 29532034 PMC5842040

[B25] Huyghues-Despointes B. M. Scholtz J. M. Pace C. N. (1999). Protein conformational stabilities can be determined from hydrogen exchange rates. Nat. Struct. Biol. 6, 910–912. 10.1038/13273 10504722

[B26] Ioannidis N. M. Rothstein J. H. Pejaver V. Middha S. Mcdonnell S. K. Baheti S. (2016). Revel: An ensemble method for predicting the pathogenicity of rare missense variants. Am. J. Hum. Genet. 99, 877–885. 10.1016/j.ajhg.2016.08.016 27666373 PMC5065685

[B27] James Z. M. Zagotta W. N. (2018). Structural insights into the mechanisms of CNBD channel function. J. Gen. Physiol. 150, 225–244. 10.1085/jgp.201711898 29233886 PMC5806680

[B28] Jiang Q. Li K. Lu W. J. Li S. Chen X. Liu X. J. (2018). Identification of small-molecule ion channel modulators in *C. Elegans* channelopathy models. Nat. Commun. 9, 3941. 10.1038/s41467-018-06514-5 30258187 PMC6158242

[B29] Kamisetty H. Ovchinnikov S. Baker D. (2013). Assessing the utility of coevolution-based residue-residue contact predictions in a sequence- and structure-rich era. Proc. Natl. Acad. Sci. U. S. A. 110, 15674–15679. 10.1073/pnas.1314045110 24009338 PMC3785744

[B30] Kanner S. A. Jain A. Colecraft H. M. (2018). Development of A high-throughput flow cytometry assay to monitor defective trafficking and rescue of long QT2 mutant hERG channels. Front. Physiol. 9, 397. 10.3389/fphys.2018.00397 29725305 PMC5917007

[B31] Kozek K. A. Glazer A. M. Ng C. A. Blackwell D. Egly C. L. Vanags L. R. (2020). High-throughput discovery of trafficking-deficient variants in the cardiac potassium channel Kv11.1. Heart Rhythm 17, 2180–2189. 10.1016/j.hrthm.2020.05.041 32522694 PMC7704534

[B32] Landrum M. J. Chitipiralla S. Brown G. R. Chen C. Gu B. S. Hart J. (2020). ClinVar: Improvements to accessing data. Nucleic Acids Res. 48, D835–D844. 10.1093/nar/gkz972 31777943 PMC6943040

[B33] Landrum M. J. Lee J. M. Riley G. R. Jang W. Rubinstein W. S. Church D. M. (2014). Clinvar: Public archive of relationships among sequence variation and human phenotype. Nucleic Acids Res. 42, D980–D985. 10.1093/nar/gkt1113 24234437 PMC3965032

[B34] Lee S. Y. Banerjee A. Mackinnon R. (2009). Two separate interfaces between the voltage sensor and pore are required for the function of voltage-dependent K^+^ channels. PLoS Biol. 7, E47. 10.1371/journal.pbio.1000047 19260762 PMC2650729

[B35] Leong I. U. Stuckey A. Lai D. Skinner J. R. Love D. R. (2015). Assessment of the predictive accuracy of five *in silico* prediction tools, alone or in combination, and two metaservers to classify long QT syndrome gene mutations. BMC Med. Genet. 16, 34. 10.1186/s12881-015-0176-z 25967940 PMC4630850

[B36] Li X. Martinson A. S. Layden M. J. Diatta F. H. Sberna A. P. Simmons D. K. (2015). Ether-A-go-go family voltage-gated K^+^ channels evolved in an ancestral metazoan and functionally diversified in a Cnidarian-bilaterian ancestor. J. Exp. Biol. 218, 526–536. 10.1242/jeb.110080 25696816 PMC4334144

[B37] Lin E. C. Moungey B. M. Balijepalli S. Y. January C. T. (2011). Different molecular phenotypes of LQT2-linked hERG1a mutations in the same amino acid. Biophysical J. 100, 30. 10.1016/j.bpj.2010.12.366

[B38] Liu L. Hayashi K. Kaneda T. Ino H. Fujino N. Uchiyama K. (2013). A novel mutation in the transmembrane nonpore region of the KCNH2 gene causes severe clinical manifestations of long QT syndrome. Heart Rhythm 10, 61–67. 10.1016/j.hrthm.2012.09.053 23010577

[B39] Marinko J. T. Huang H. Penn W. D. Capra J. A. Schlebach J. P. Sanders C. R. (2019). Folding and misfolding of human membrane proteins in health and disease: From single molecules to cellular proteostasis. Chem. Rev. 119, 5537–5606. 10.1021/acs.chemrev.8b00532 30608666 PMC6506414

[B40] Napolitano C. Priori S. G. Schwartz P. J. Bloise R. Ronchetti E. Nastoli J. (2005). Genetic testing in the long QT syndrome: Development and validation of an efficient approach to genotyping in clinical practice. JAMA 294, 2975–2980. 10.1001/jama.294.23.2975 16414944

[B41] Ng C. A. Perry M. D. Liang W. Smith N. J. Foo B. Shrier A. (2020). High-throughput phenotyping of heteromeric human ether-A-go-go-related gene potassium channel variants can discriminate pathogenic from rare benign variants. Heart Rhythm 17, 492–500. 10.1016/j.hrthm.2019.09.020 31557540

[B42] Ng C. A. Ullah R. Farr J. Hill A. P. Kozek K. A. Vanags L. R. (2022). A massively parallel assay accurately discriminates between functionally normal and abnormal variants in a hotspot domain of *KCNH2* . Am. J. Hum. Genet. 109, 1208–1216. 10.1016/j.ajhg.2022.05.003 35688148 PMC9300756

[B43] Nicoludis J. M. Gaudet R. (2018). Applications of sequence coevolution in membrane protein biochemistry. Biochim. Biophys. Acta. Biomembr. 1860, 895–908. 10.1016/j.bbamem.2017.10.004 28993150 PMC5807202

[B44] Oliveira-Mendes B. Feliciangeli S. Menard M. Chatelain F. Alameh M. Montnach J. (2021). A standardised hERG phenotyping pipeline to evaluate *KCNH2* genetic variant pathogenicity. Clin. Transl. Med. 11, E609. 10.1002/ctm2.609 34841674 PMC8609418

[B45] Palovcak E. Delemotte L. Klein M. L. Carnevale V. (2014). Evolutionary imprint of activation: The design principles of VSDs. J. Gen. Physiol. 143, 145–156. 10.1085/jgp.201311103 24470486 PMC4001776

[B46] Perry M. D. Ng C. A. Phan K. David E. Steer K. Hunter M. J. (2016). Rescue of protein expression defects may not be enough to abolish the pro-arrhythmic phenotype of long QT type 2 mutations. J. Physiol. 594, 4031–4049. 10.1113/JP271805 26958806 PMC4945714

[B47] Phan K. Ng C. A. David E. Shishmarev D. Kuchel P. W. Vandenberg J. I. (2017). The S1 helix critically regulates the finely tuned gating of Kv11.1 channels. J. Biol. Chem. 292, 7688–7705. 10.1074/jbc.M117.779298 28280240 PMC5418064

[B48] Phul S. Kuenze G. Vanoye C. G. Sanders C. R. George A. L. Jr. Meiler J. (2022). Predicting the functional impact of KCNQ1 variants with artificial neural networks. PLoS Comput. Biol. 18, E1010038. 10.1371/journal.pcbi.1010038 35442947 PMC9060377

[B49] Schlebach J. P. Narayan M. Alford C. Mittendorf K. F. Carter B. D. Li J. (2015). Conformational stability and pathogenic misfolding of the integral membrane protein PMP22. J. Am. Chem. Soc. 137, 8758–8768. 10.1021/jacs.5b03743 26102530 PMC4507940

[B50] Shelar A. Bansal M. (2016). Helix perturbations in membrane proteins assist in inter-helical interactions and optimal helix positioning in the bilayer. Biochim. Biophys. Acta 1858, 2804–2817. 10.1016/j.bbamem.2016.08.003 27521749

[B51] Sun J. Mackinnon R. (2020). Structural basis of human KCNQ1 modulation and gating. Cell. 180, 340–347. 10.1016/j.cell.2019.12.003 31883792 PMC7083075

[B52] Taliun D. Harris D. N. Kessler M. D. Carlson J. Szpiech Z. A. Torres R. (2021). Sequencing of 53,831 diverse genomes from the NHLBI topmed program. Nature 590, 290–299. 10.1038/s41586-021-03205-y 33568819 PMC7875770

[B53] Tanaka T. Nagai R. Tomoike H. Takata S. Yano K. Yabuta K. (1997). Four novel KvLQT1 and four novel hERG mutations in familial long-QT syndrome. Circulation 95, 565–567. 10.1161/01.cir.95.3.565 9024139

[B54] Vanoye C. G. Desai R. R. Fabre K. L. Gallagher S. L. Potet F. Dekeyser J. M. (2018). High-throughput functional evaluation of KCNQ1 decrypts variants of Unknown significance. Circ. Genom. Precis. Med. 11, E002345. 10.1161/CIRCGEN.118.002345 30571187 PMC6309341

[B55] Walsh R. Peters N. S. Cook S. A. Ware J. S. (2014). Paralogue annotation identifies novel pathogenic variants in patients with brugada syndrome and catecholaminergic polymorphic ventricular tachycardia. J. Med. Genet. 51, 35–44. 10.1136/jmedgenet-2013-101917 24136861 PMC3888601

[B56] Wang Q. L. Dhindsa R. S. Carss K. Harper A. R. Nag A. Tachmazidou I. (2021). Rare variant contribution to human disease in 281,104 UK biobank exomes. Nature 597, 527–532. 10.1038/s41586-021-03855-y 34375979 PMC8458098

[B57] Wang W. Mackinnon R. (2017). Cryo-EM structure of the open human ether-A-go-go-related K^+^ channel hERG. Cell. 169, 422–430. 10.1016/j.cell.2017.03.048 28431243 PMC5484391

[B58] Yampolsky L. Y. Stoltzfus A. (2005). The exchangeability of amino acids in proteins. Genetics 170, 1459–1472. 10.1534/genetics.104.039107 15944362 PMC1449787

[B59] Zhang J. Kim E. C. Chen C. Procko E. Pant S. Lam K. (2020). Identifying mutation hotspots reveals pathogenetic mechanisms of KCNQ2 epileptic encephalopathy. Sci. Rep. 10, 4756. 10.1038/s41598-020-61697-6 32179837 PMC7075958

[B60] Zhang Y. Colenso C. K. El Harchi A. Cheng H. Witchel H. J. Dempsey C. E. (2016). Interactions between amiodarone and the hERG potassium channel pore determined with mutagenesis and *in silico* docking. Biochem. Pharmacol. 113, 24–35. 10.1016/j.bcp.2016.05.013 27256139 PMC4959829

[B61] Zhang Y. H. Colenso C. K. Sessions R. B. Dempsey C. E. Hancox J. C. (2011). The hERG K^+^ channel S4 domain L532P mutation: Characterization at 37°C. Biochim. Biophys. Acta 1808, 2477–2487. 10.1016/j.bbamem.2011.07.001 21777565 PMC3245891

[B62] Zhou Z. Gong Q. January C. T. (1999). Correction of defective protein trafficking of a mutant hERG potassium channel in human long QT syndrome. Pharmacological and temperature effects. J. Biol. Chem. 274, 31123–31126. 10.1074/jbc.274.44.31123 10531299

