# Supplementary material for: Evolutionary coupling analysis guides identification of mistrafficking-sensitive variants in cardiac K+ channels: Validation with hERG
Source: Front Pharmacol. 2022 Oct 20;13:1010119. doi: 10.3389/fphar.2022.1010119 (PMC9632996; doi:10.3389/fphar.2022.1010119)
Supplement: Supplementary file 3 [file DataSheet1.DOCX]

**SUPPLEMENTARY INFORMATION for:**

**Evolutionary Coupling Analysis Guides Identification of Mistrafficking-Sensitive Variants in Cardiac K+ Channels: Validation with hERG**

*Yihong Zhang, Amy L. Grimwood, Jules C. Hancox, Stephen C. Harmer* and Christopher E. Dempsey**

This document contains Supplementary Tables S1 - S4 and Supplementary Figures S1 – S3 referred to in the main text. Citations refer to references in the main paper. The contents of the additional Supplementary Excel Data Files are described below:

**Description of Additional Supplementary Excel Data Files**

**File name: Table 1.xlsx:**

**Description:** Series of EC analyses obtained from EVcouplings queries containing the KCNQ1 VSD sequence that was used for Table 1 of the main paper. A key to the data is below Table column AL.

**File name: Table 2.xlsx:**

**Description:** Series of EC analyses obtained from EVcouplings queries for hERG.

***Sheet 1:*** Series of EC analyses obtained from queries containing hERG VSD S2-S3 loop sequence used for choosing variants listed in Table 3 of the main paper. A description of the data is provided in the text box below column S.

***Sheet 2:*** Similar analysis to identify EC pairs in the hERG pore turret involving the turret loop (residues 593-606). A description of the data is provided in the text box below column B.

***Sheet 3:*** A comparison of trafficking data for hERG membrane domain variants with EC that was used for Table 2 of the main paper. A description of the data is provided in the text box below column S.

| ***KCNH2 A*** | ***hERG_Nterm*** | ***hERG_mem*** | ***hERG_Cterm*** |
| --- | --- | --- | --- |
| total variants | 705 | 488 | 819 |
| synonymous | 263 | 142 | 268 |
| frame shift | 58 | 24 | 128 |
| stop codon | 18 | 17 | 24 |
| deletions | 2 | 2 | 2 |
| total missense | 364 | 301 | 398 |

| ***KCNH2 B*** | ***hERG_Nterm*** | ***hERG_mem*** | ***hERG_Cterm*** |
| --- | --- | --- | --- |
| total missense | 364 | 301 | 398 |
| benign | 2 | 1 | 3 |
| benign/likely benign | 0 | 0 | 4 |
| conflicting interpret | 23 | 17 | 34 |
| likely benign | 19 | 1 | 9 |
| likely pathogenic | 22 | 42 | 11 |
| not provided | 60 | 94 | 33 |
| pathogenic | 10 | 44 | 7 |
| pathogenic/likely path | 5 | 10 | 10 |
| uncertain significance | 223 | 92 | 287 |
| % uncharacterized | 84% | 67% | 89% |

| ***KCNQ1 A*** | ***KCNQ1_Nterm*** | ***KCNQ1_mem*** | ***KCNQ1_Cterm*** |
| --- | --- | --- | --- |
| total variants | 95 | 634 | 598 |
| synonymous | 32 | 243 | 269 |
| frame shift | 9 | 34 | 48 |
| stop codon | 7 | 22 | 16 |
| Deletions | 0 | 8 | 2 |
| total missense | 47 | 338 | 263 |

| ***KCNQ1 B*** | ***KCNQ1_Nterm*** | ***KCNQ1_mem*** | ***KCNQ1_Cterm*** |
| --- | --- | --- | --- |
| total missense | 47 | 338 | 263 |
| benign | 0 | 0 | 1 |
| benign/likely benign | 0 | 0 | 4 |
| conflicting interpret | 1 | 30 | 29 |
| likely benign | 1 | 1 | 3 |
| likely pathogenic | 1 | 40 | 14 |
| not provided | 4 | 65 | 29 |
| pathogenic | 2 | 68 | 14 |
| pathogenic/likely path | 0 | 21 | 12 |
| uncertain significance | 38 | 113 | 157 |
| % uncharacterized | 91% | 62% | 82% |

***Supplementary Table S1:*** Numbers of KCNH2 and KCNQ1 genetic variants compiled in ClinVar (assessed on 29^th^ Jan 2022) and separated into the N-terminal, membrane and C-terminal channel domains. In block A the variants are defined according to their genetic type and in Block B the missense variants are further grouped according to ClinVar classifications. Conflicting interpret: conflicting interpretation; likely path: likely pathogenic. The % uncharacterized is the percentage of variants in “conflicting interpretation” + “not provided” + “uncertain significance” categories.

| ***EC mutant*** | ***Nucleotide variation*** | ***Forward primers*** | ***Reverse primers*** |
| --- | --- | --- | --- |
| I400N | 1199T>A | CCACCGCTGGACCAACCTGCATTACAG | CTGTAATGCAGGTTGGTCCAGCGGTGG |
| H402R | 1205A>G | CTGGACCATCCTGCGTTACAGCCCCTTC | GAAGGGGCTGTAACGCAGGATGGTCCAG |
| K407A | Not in ClinVar | CAGCCCCTTCGCGGCCGTGTGG | CCACACGGCCGCGAAGGGGCTG |
| R472P | 1415G>C | CTCATCAACTTCCCCACCACCTACGTC | GACGTAGGTGGTGGGGAAGTTGATGAG |
| T473P | 1417A>C | CCTCATCAACTTCCGCCCCACCTACGTCAATGC | GCATTGACGTAGGTGGGGCGGAAGTTGATGAGG |
| T474I | 1421C>T | CATCAACTTCCGCACCATCTACGTCAATGCCAACG | CGTTGGCATTGACGTAGATGGTGCGGAAGTTGATG |
| Y475C | 1424A>G | CTTCCGCACCACCTGCGTCAATGCCAAC | GTTGGCATTGACGCAGGTGGTGCGGAAG |
| V476I | 1426G>A | GCACCACCTACATCAATGCCAACG | CGTTGGCATTGATGTAGGTGGTGC |
| V483F | 1447G>T | CGAGGAGGTGTTCAGCCACCC | GGGTGGCTGAACACCTCCTCG |
| R488C | 1462C>T | CACCCCGGCTGCATCGCCGTC | GACGGCGATGCAGCCGGGGTG |
| H492L | 1475A>T | GCATCGCCGTCCTCTACTTCAAGG | CCTTGAAGTAGAGGACGGCGATGC |
| A614V | 1841C>T | GACAAGTATGTGACGGTGCTCTACTTCACCTTC | GAAGGTGAAGTAGAGCACCGTCACATACTTGTC |
| L615F | 1843C>T | GTATGTGACGGCGTTCTACTTCACCTTC | GAAGGTGAAGTAGAACGCCGTCACATAC |

***Supplementary Table S2:*** Primers used to generate hERG membrane domain genetic variants.

| ***Channel domain*** | ***Approximate sequence range in structure^1^*** | ***Sequence range submitted to EVcouplings server^2^*** |
| --- | --- | --- |
| **KCNQ1** | **(PDB:6V00)** |  |
| membrane | T104 – L353 | Y94 – R360 |
| pore | - | - |
| VSD | T104 – G245 | Y94 – V255 |
| **hERG (KCNH2)** | **(PDB:5VA2)** |  |
| membrane | W398 – Y667 | L390 – A671 |
| pore^3^ | G546 – Y667 | D540 – A671 |
| VSD^3^ | W398 – R541 | L390 – G546 |

***Supplementary Table S3:*** Amino acid sequence ranges used to identify high-probability EC interactions in KCNQ1 and hERG membrane domains.

^1^For KCNQ1 and hERG, atom density is missing for membrane domain sequence before residues T104 (KCNQ1; PDB: 6V00) and W398 (hERG; PDB: 5VA2) so that these residues define the N-terminal limit of the structures of membrane and VSD domains for the respective channels.

^2^Submitted ranges were extend by up to 10 residues at the N- and C-termini to maximise recovery of high-probability EC interactions involving residues near the termini of regions of interest.

^3^Additional sequences ranges were submitted for the VSD and pore domains of hERG to assess reproducibility of high-probability EC’s in the hERG VSD S2-S3 loop (supplementary Table 2.xlsx, sheet 1 and Supplementary Figure S1) and hERG pore turret loop (supplementary Table 2.xlsx, sheet 2).

| **Variants**  **[In ClinVar]**  **(repeats)** | **Cell surface channel expression**  **(% of WT)**  **‘On-Cell’ assay** | **Total channel detected**  **(% of WT)**  **‘In-Cell’ assay** | **Apparent trafficking efficiency**  **(% of WT)** |
| --- | --- | --- | --- |
| **A614V**  [pathogenic] (8) | 8.8±0.5 | 89.9±2.9 | 9.9±0.6 |
| **L615F**  [pathogenic] (12) | 4.8±0.7 | 89.9±5.0 | 5.2±0.6 |
| **I400N**  [VUS] (4) | 6.1±1.1 | 80.0±1.2 | 7.6±1.4 |
| **H402R**  [VUS)] (4) | 13.0±1.5 | 87.3±6.5 | 15.0±1.7 |
| **K407A**  [not in Clinvar] (4) | 114.7±12.1 | 103.0±8.5 | 111.1±6.1 |
| **R472P**  [likely pathogenic] (4) | 0.9±0.5 | 79.1±6.9 | 1.1±0.7 |
| **T473P**  [VUS] (4) | 1.9±0.7 | 76.7±6.1 | 2.4±0.8 |
| **T474I**  [pathogenic] (4) | 10.4±.1.2 | 82.9±2.6 | 12.5±1.2 |
| **Y475C**  [VUS] (4) | 11.0±1.4 | 77.0±3.4 | 14.3±14.3 |
| **V476I**  [VUS] (4) | 84.9±6.8 | 91.0±4.3 | 93.1±5.3 |
| **V483F**  [VUS] (4) | 3.1±0.8 | 64.9±6.5 | 5.1±1.4 |
| **R488C**  [VUS] (4) | 68.3±4.3 | 78.0±8.9 | 89.1±6.0 |
| **H492L**  [VUS] (4) | 18.9±2.7 | 65.7±4.8 | 28.3±2.4 |

***Supplementary Table S4*: Apparent ‘trafficking efficiency’ for hERG voltage sensor domain variants and controls.**

The apparent trafficking efficiency (compared to WT) was calculated using the approach and equation described in Huang *et al*. (2018):

‘Apparent trafficking efficiency’ (in %) = [(Cell Surface expression)Variant/(Total protein detected)Variant]/[( Cell Surface expression)WT/(Total protein detected)WT] × 100.

Data are presented as mean ± SEM. VUS: Variant of uncertain significance. The value in parenthesis in column one is the number of independent replicates. A graphical representation and statistical analysis of the ratio value generated upon Cell Surface expression normalisation to Total protein detected is provided in Supplementary Figure S2. The first two columns are the same as presented in Table 4 of the main paper.


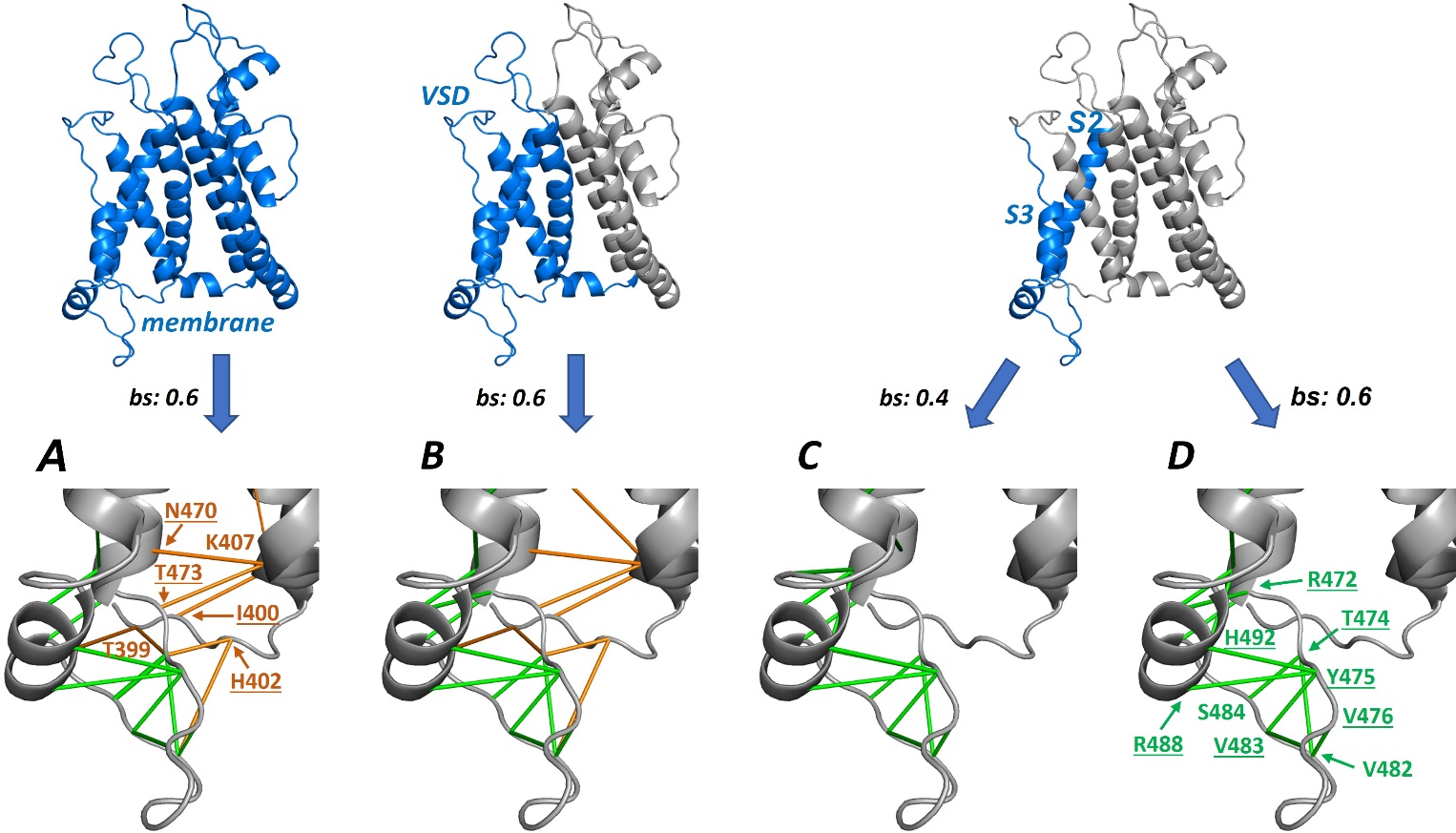


***Supplementary Figure S1.*** Identification of reproducible Evolutionary Coupled pairs of amino acids in and around the hERG VSD S2-S3 loop for selecting variants for experimental trafficking. Underlined residues are amino acids that are *both* part of an EC pair *and* have at least one variant reported in ClinVar. Apart from N470 these are the amino acids whose ClinVar variants were selected for trafficking assay; the trafficking properties of the N470D variant are already well characterised (Zhou et al., 1999) and this residue wasn’t selected.

In this example, four EC data sets obtained from the EVcouplings server used the sequence and Bitscore (bs) submissions listed below (A-D). The sequence ranges are illustrated (blue backbone cartoon) in the top row, mapped onto the hERG membrane domain A subunit (from PDB:5VA2). Note that the membrane domain in cryoEM structures of hERG extends from W398 to around Q667. Sequence ranges submitted to the EVcouplings server were extended at the N- and C- termini by up to 10 amino acids to maximise identification of high-probability EC’s near sequence termini (see for example Supplementary Table S3). EC pairs represented by green lines were identified in all four datasets. Brown EC’s involve amino acids that lie outside the S2-S3 sequence and so can only observed in EC datasets obtained either from the full membrane domain (A) or the VSD (B).

1. Full membrane domain: sequence 390-672; Bitscore 0.6
2. Voltage sensor domain: sequence 390-546; Bitscore 0.6
3. S2-S3 domain: sequence 452-510; Bitscore 0.4
4. S2-S3 domain: sequence 452-510; Bitscore 0.6


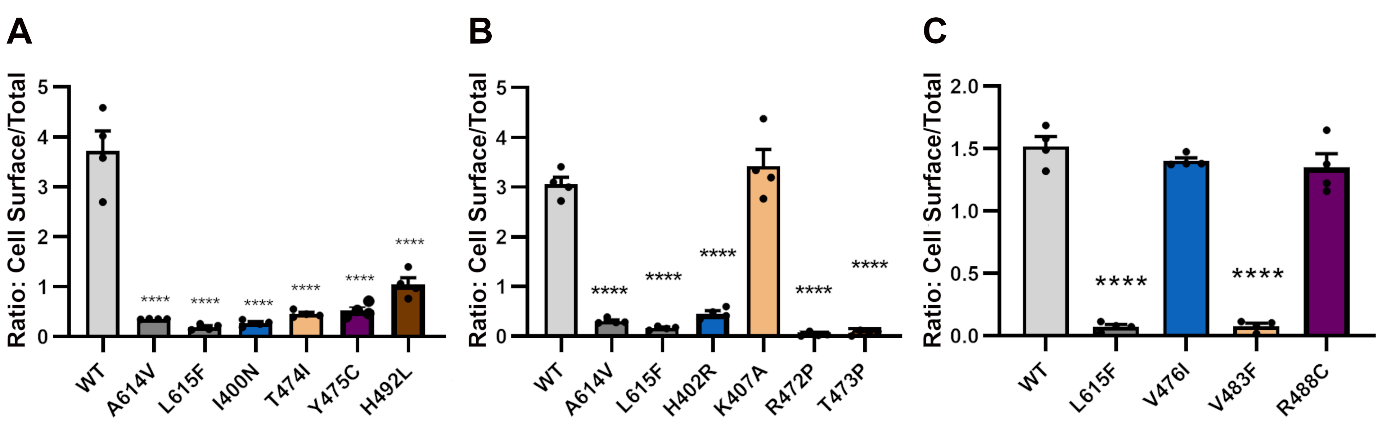


***Supplementary Figure S2:*** Comparison of the Cell Surface expression level upon normalisation to the corresponding level of Total channel protein. The values presented are the ratio of Cell Surface/Total channel protein. The ratios are generated from the On-Cell (Cell Surface) and In-Cell (Total) assays presented in Figure 5 (Panel A), Figure 6 (Panel B) and Figure 7 (Panel C) in the main paper. These ratios are used to calculate the ‘apparent trafficking efficiency’ for each variant and this is presented in Supplementary Table S4. Data are presented as (mean ± SEM) from four independent repeats. Statistical analyses were performed using one way analysis of variance (ANOVA) and Bonferroni’s multiple comparison. Asterisks indicate a signiﬁcant difference from the corresponding WT ratio value (**** = *P* < 0.0001).


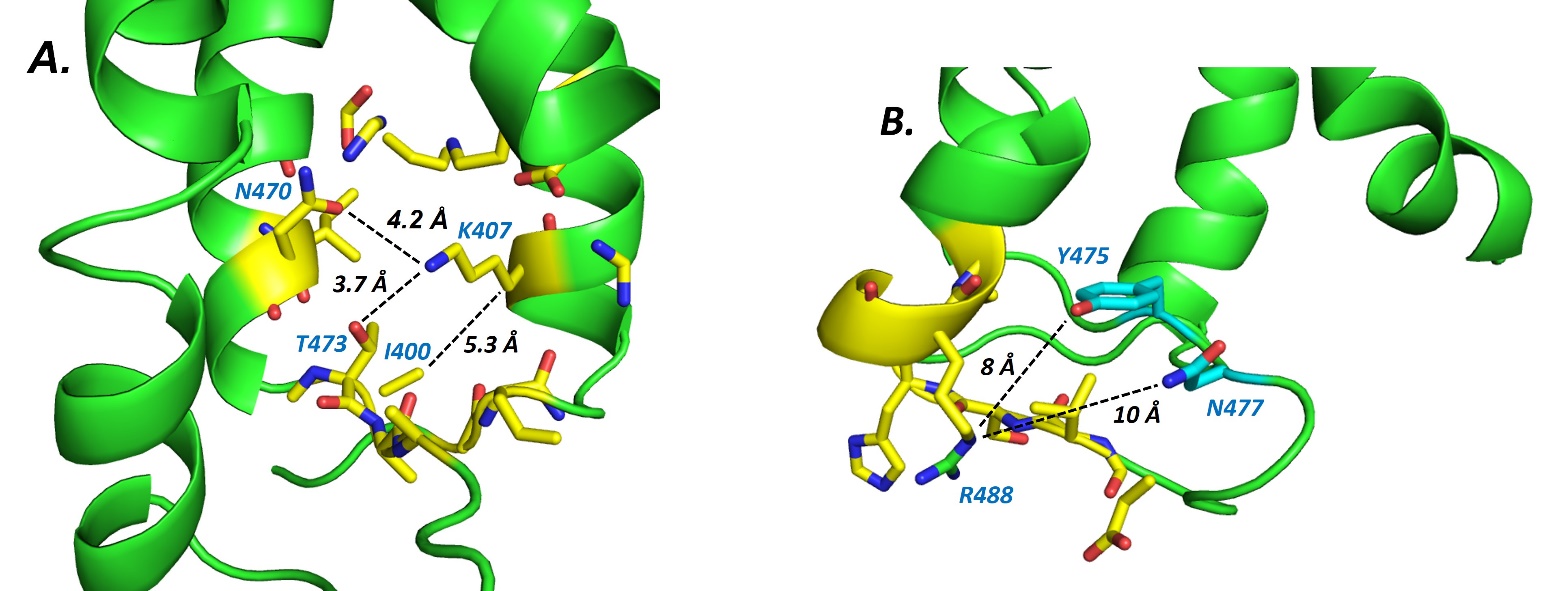


***Supplementary Figure S3:*** Structural context (PDB:5VA2) of hERG VSD residues K407 and R488 whose tested variants retain high (K407A) or moderate (R488C) trafficking efficiency. Yellow sticks are atoms within 8 Å of (***A.***) the terminal amino nitrogen of the K407 side chain and (***B.***) the central carbon of the Arg guanidine group. ***A.*** The K407 side chain interacts with residues with which it forms evolutionarily coupled pairs with favourable geometries (N470 and T473; the K407-I400 non-polar interaction is somewhat longer than ideal since the distance between carbon atoms of non-polar amino acids and the aliphatic carbons of the Lys side chain should be within around 4 Å; Armstrong et al. 2016). ***B.*** The R488 side chain guanidine group is distant from its evolutionarily coupled partners (Y475 and N477) in the cryo-EM structure; reorientation of the side chain can bring the guanidine group into a location where geometrically favourable interactions with Y475 and N477 side chains are possible. Favourable geometries for lysine and arginine side chain interactions can be found in Armstrong et al. (2016).
